# Regional-based within-year seasonal variations in influenza-related health outcomes across mainland China: a systematic review and spatio-temporal analysis

**DOI:** 10.1186/s12916-022-02269-5

**Published:** 2022-02-10

**Authors:** Charlie Diamond, Hui Gong, Fiona Yueqian Sun, Yang Liu, Billy J. Quilty, Mark Jit, Juan Yang, Hongjie Yu, W. John Edmunds, Marc Baguelin

**Affiliations:** 1 Department of Infectious Disease Epidemiology, Centre for Mathematical Modelling of Infectious Diseases, London School of Hygiene and Tropical Medicine, London, UK; 2 School of Public Health, Fudan University, Key Laboratory of Public Health Safety, Ministry of Education, Shanghai, China; 3 MRC Centre for Global Infectious Disease Analysis, School of Public Health, Imperial College London, London, UK

**Keywords:** China, Influenza, Seasonality, Systematic review, Spatio-temporal

## Abstract

**Background:**

China experiences large variations in influenza seasonal activity. We aim to update and improve the current understanding of regional-based within-year variations of influenza activity across mainland China to provide evidence for the planning and optimisation of healthcare strategies.

**Methods:**

We conducted a systematic review and spatio-temporal meta-analysis to assess regional-based within-year variations of ILI outpatient consultation rates, influenza test positivity rates amongst both ILI outpatients and SARI inpatients, and influenza-associated excess mortality rates. We searched English and Chinese databases for articles reporting time-series data on the four influenza-related outcomes at the sub-national and sub-annual level. After synthesising the data, we reported on the mean monthly rate, epidemic onset, duration, peak and intensity.

**Results:**

We included 247 (7.7%) eligible studies in the analysis. We found within-year influenza patterns to vary across mainland China in relation to latitude and geographic location. High-latitude provinces were characterised by having short and intense annual winter epidemics, whilst most mid-latitude and low-latitude provinces experience semi-annual epidemics or year-round activity. Subtype activity varied across the country, with A/H1N1pdm09 and influenza B occurring predominantly in the winter, whereas A/H3N2 activity exhibited a latitudinal divide with high-latitude regions experiencing a winter peak, whilst mid and low-latitude regions experienced a summer epidemic. Epidemic onsets and peaks also varied, occurring first in the north and later in the southeast. We found positive associations between all influenza health outcomes. In addition, seasonal patterns at the prefecture and county-level broadly resembled their wider province.

**Conclusions:**

This is the first systematic review to simultaneously examine the seasonal variation of multiple influenza-related health outcomes at multiple spatial scales across mainland China. The seasonality information provided here has important implications for the planning and optimisation of immunisation programmes and healthcare provision, supporting the need for regional-based approaches to address variations in local epidemiology.

**Supplementary Information:**

The online version contains supplementary material available at 10.1186/s12916-022-02269-5.

## Background

Each year, seasonal influenza is responsible for a substantial burden of disease [1–3]. With a total population in excess of 1.4 billion people, mainland China experiences a large influenza morbidity and mortality burden [4]. Each year, it is estimated there are approximately 2.5 influenza-associated influenza-like-illness (ILI) consultations per 1000 people and between 84,200 and 92,000 influenza-associated excess respiratory deaths [5, 6]. Despite this, influenza vaccines are not currently covered by the government-funded National Immunisation Program (NIP) for most of the population in mainland China. Hence, the cost is usually borne by the consumer, resulting in low uptake [7]. In recent years, some wealthier local governments have offered free influenza vaccination to older adults, leading to increased coverage [8–10].

Influenza seasonality varies substantially both between and within countries [11]. In non-pandemic periods, seasonal influenza epidemics are characterised by strong winter peaks in temperate high and low-latitude regions. Conversely, sub-tropical and tropical regions broadly experience semi-annual influenza peaks or year-round activity [11–13]. Mainland China spans a vast geographic area and experiences large variations in influenza seasonality. High-latitude provinces have been shown to exhibit influenza seasons similar to those of other temperate regions in the northern hemisphere, whilst mid and low-latitude provinces experience a less pronounced seasonal burden, more reflective of tropical and subtropical regions [14–16].

Whilst many studies have estimated the burden of influenza-related health outcomes across mainland China, results are often reported at the annual level, neglecting the within-year variations [5, 6]. Meanwhile, studies that examine the within-year seasonal variation of influenza are usually limited to an individual region [17–20], or only report on one specific outcome e.g. influenza test positivity rates [14]. Some studies only report on a single influenza season, in a single setting, making it difficult to contextualise both spatially or temporally [21, 22]. Whilst all these studies provide useful elements individually, we aim to synthesise all the available information from both the English and Chinese literature to update and improve the current characterisation of the within-year seasonal variation of multiple influenza health outcomes across mainland China, at multiple spatial scales. In turn, this will provide evidence for the planning and optimisation of influenza immunisation programmes and public health strategies across mainland China.

## Methods

### Search strategy and selection criteria

We collected time-series data on four key influenza-related health outcomes across mainland China through a systematic search of both the English and Chinese literature. We selected outcomes to capture all levels of disease severity, this included (1) ILI outpatient consultation rates; (2) influenza test positivity rates amongst both ILI outpatients; (3) severe acute respiratory infection (SARI) inpatients; and (4) influenza-associated excess mortality rates. We further included strain-specific test positivity rates for the most common seasonal influenza strains as a sub-category for the overall test positive rates (i.e. under (2)). This included A/H3N2, A/H1N1pdm09 and influenza B viruses.

We followed the Preferred Reporting Items for Systematic Reviews and Meta-Analyses (PRISMA) guidelines [23] and searched four databases (PubMed, Web of Science, WanFang and CNKI) for English or Chinese articles reporting time-series data on any of the above outcomes. The specific search terms included “influenza”, “ILI”, “test positive”, “hospitalisation, “mortality”, “China”, “time-series” and “seasonality”, with many other closely related words and synonyms along with their Chinese translations (Additional file 1: Appendix 1). We limited the literature search to articles released between January 1, 2000, to September 31, 2019, as prior to this there was a lack of consistent laboratory surveillance data widely available across mainland China [14]. Additionally, we aimed to provide a snapshot of the current state of influenza seasonality within China based on recent years; any available historic data prior to this is less likely to be relevant to current seasonality patterns. Each article identified through the search strategy went through a process of title, abstract and full-text screening based upon a set of inclusion and exclusion criteria (Additional file 1: Appendix 2). To ensure consistency between reviewers, we conducted a blind screening on a random sample of 200 articles identified by the search strategy. In this exercise, authors had a high level of agreement between studies (>95%), and any conflicts were discussed and resolved.

### Data extraction

WebPlotDigitizer (version 4.2 )[24] was used to extract data from figures, the R package tabulizer [25] was used to extract data from tables, and we also collected data from studies where the raw data was publicly available. All extracted data was cleaned and is available on GitHub (https://github.com/EIDECDIA/China_influenza_seasonality). See Additional file 1: Appendix 3 for further details on how data was processed and cleaned.

### Data analysis

#### Mean monthly influenza activity

To estimate the within-year seasonal characteristics of an influenza-related health outcome, we first calculated the mean monthly rate (MMR) in each administrative region (province, prefecture and county) with available data (Additional file 1: Appendix 4). The MMR captures the average multi-year seasonal trend of influenza health outcomes in a given setting. For instance, the MMR of influenza test positivity can be interpreted as the average proportion of influenza tests reporting positive results in any given month. We calculated the MMR separately for both pre and post-2009/2010 influenza pandemic periods, whilst excluding the impact of the pandemic period itself. We further defined an epidemic’s average peak and low point as months with the maximum and minimum MMR of influenza test positivity among ILI outpatients.

#### Epidemic onset and duration

We estimated the average relative duration of an influenza epidemic in a given region based upon the proportion of the total average annual influenza test positivity among ILI outpatients (MMR) which occurred during each month of the year, as seen in Li et al (2019) [11] (Additional file 1: Appendix 5). The minimum number of non-consecutive months needed to cross an arbitrary relative threshold of >75% of the total annual activity were defined as *“Epidemic”* months, whilst the remainder were denoted *“Non-epidemic”* months. The frequency of *“Epidemic”* months in a year gave the average epidemic duration, and the first *“Epidemic”* month following the start of the influenza epidemiological year (defined here as October) was classed as the *“Epidemic onset”* month, aligning the influenza seasonality pattern more broadly with the northern hemisphere.

#### Epidemic intensity

To estimate the relative intensity of influenza epidemics we used a measure adapted from Dalziel et al (2018) [26], where epidemic intensity is based upon the inverse of the Shannon entropy of influenza activity distribution in a given region and year (Additional file 1: Appendix 6) [27]. Thus, epidemic intensity describes how evenly distributed incidence is spread across a year and is therefore maximised when activity is concentrated to a small proportion of a year and minimised when it is evenly dispersed across it.

#### Long-term trend

We conducted a seasonal decomposition analysis to further investigate any identifiable seasonal patterns and long-term secular trends. Using a classical additive time-series decomposition model on our province-level influenza test positivity rates among ILI outpatients, we decomposed the time-series into their respective long-term trends, seasonal effects and random error components (Additional file 1: Appendix 7).

### Implementation

All data cleaning and analyses were carried out on R version 4.0.5.

## Results

### Search results

**Fig. 1:**
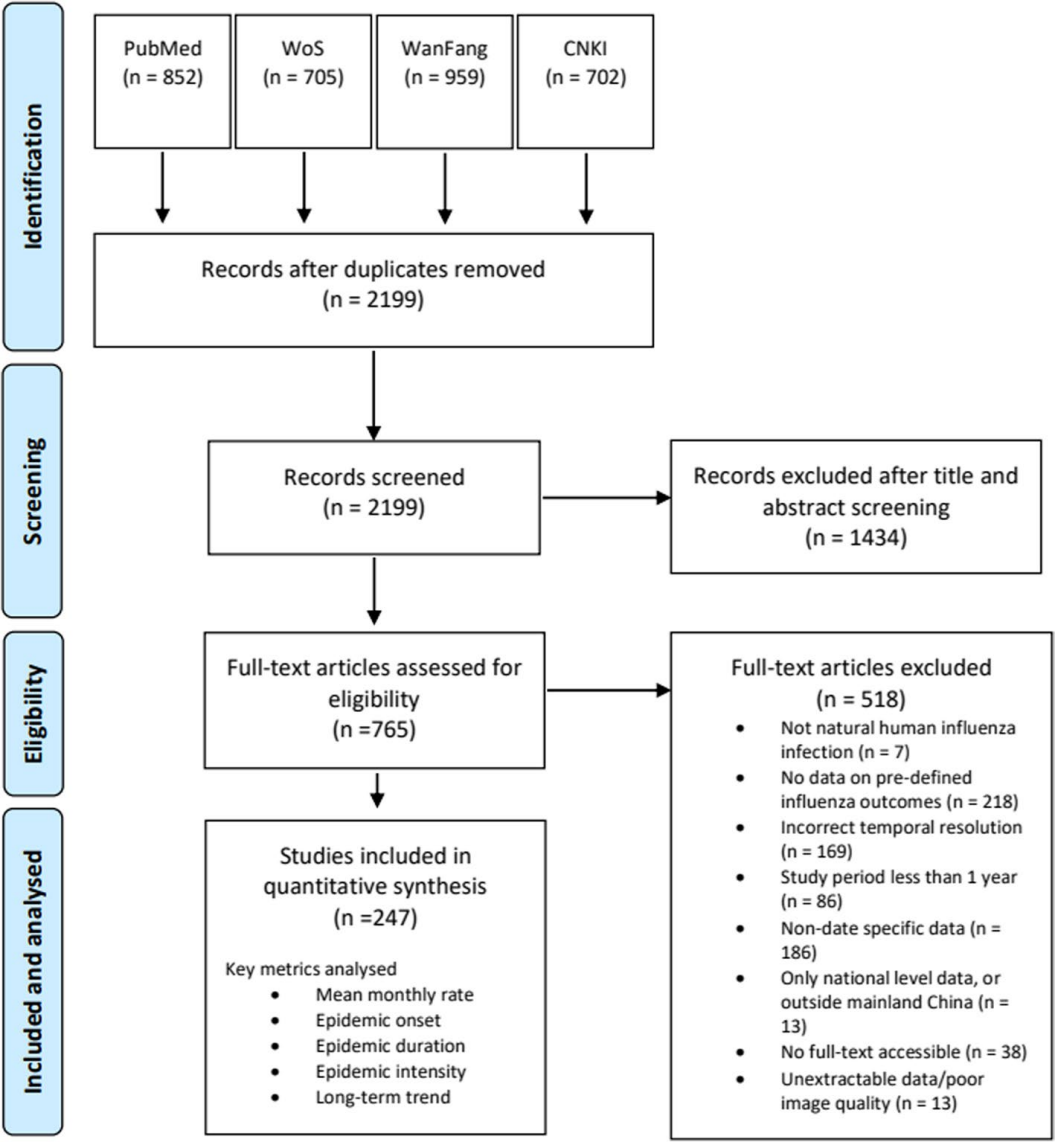
Flow chart of study selection. Studies could be excluded on multiple grounds

We identified 3218 studies through the literature search (Fig. 1). After excluding duplicates, 2199 (68.3%) remained, of which 765 (23.7%) passed the title and abstract screening, and 247 (7.7%) were identified as eligible studies containing suitable data from the full-text screening. A full list of these articles can be found in Additional file 1: Table S1.

Studies varied substantially on the basis of geographic scope, temporal resolution and the number of years data was reported. From 2009 onwards, there was a considerable increase in the number of studies published with the desired information, likely the result of increased surveillance, particularly after the 2009/2010 influenza pandemic (Additional file 1: Fig. S2). The proportion of China represented by each of the outcomes considered in this study varies dramatically, with influenza test positivity rates amongst ILI outpatients being the most widely reported indicator, followed by ILI consultation rates. Only a few studies identified reported influenza positivity rates amongst SARI inpatients and influenza-associated excess mortality rates (Table 1 and Additional file 1: Fig. S3).

**Table 1:** Number of studies reporting the specified time-series data for each influenza health outcome by administrative region. Number and percentage of administrative regions represented by eligible studies also presented

***Outcome***	***Province*** *(total = 32)*		***Prefecture*** *(total = 341)*		***County*** *(total = 2937)*	
	Number of studies	Number of provinces covered (%)	Number of studies	Number of prefectures covered (%)	Number of studies	Number of counties covered (%)
ILI consultation rate (all outpatients)	29	14 (43.8%)	72	56 (16.4%)	30	25 (0.85%)
Test positivity (ILI outpatients)	32	30 (93.8%)	92	64 (18.8%)	31	23 (0.78%)
Test positivity (SARI inpatients)	2	2 (6.25%)	2	2 (0.58%)	0	0 (0%)
Influenza excess mortality rate (per 100 000)	0	0 (0%)	3	3 (0.88%)	0	0 (0%)

Most studies focused on regions with higher GDP per capita and population, particularly in Eastern and Central China, with less populous and developed regions being underrepresented, most notably in Western China. We found the mean total population of prefectures reporting ILI consultation rate data to be 5.01 (95% CI 4.23–5.78) million people, compared to the national prefecture population mean of 3.72 million people (Additional file 1: Fig. S4). For within-year test positivity rate among ILI outpatients specifically, we identified at least one study reporting data for every province, with the exception of the Tibet Autonomous Region. Studies reporting sub-provincial estimates were well dispersed across all provinces. In contrast, ILI consultation rate data is more clustered, with less widespread provincial-level data reported. This has created a few spatial gaps (e.g. Yunnan and Qinghai) where we were not able to identify any ILI consultation rate data from any administrative level.

### Mean monthly influenza activity

**Fig. 2:**
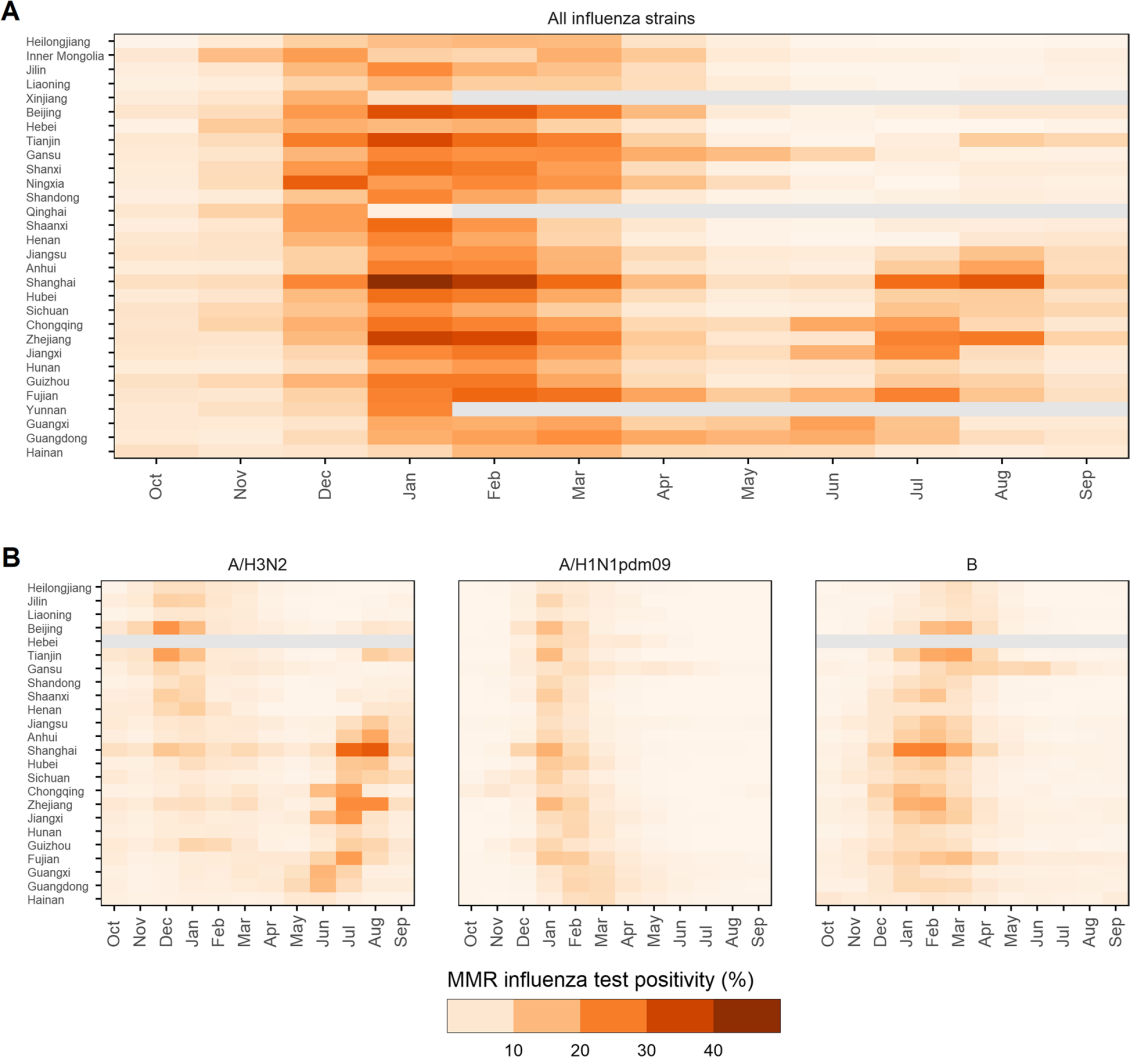
Post-pandemic provincial mean monthly rate (MMR) of influenza test positivity among ILI outpatients, sorted by descending province centroid latitude. **A** Influenza test positive rate for all common seasonal strains combined. **B** Influenza test positive rates disaggregated by A/H3N2, A/H1N1pdm09 and B subtypes.

Figure 2A displays the post-pandemic province-level MMR for all strains of influenza test-positivity, sorted by decreasing latitude. In higher-latitude provinces, most influenza activity remains constrained between December - March. The picture is less clear in mid-latitude and low-latitude provinces where semi-annual epidemics (e.g. Zhejiang and Anhui) and year-round activity (e.g. Hainan) occur. Averaged across all provinces, January was the peak month of activity with a mean positivity rate of 21.8%, ranging from 1.99% in Qinghai to 42.5% in Shanghai. In contrast, October has the lowest MMR of 4.37%, ranging from 0.41% in Heilongjiang to 7.22% in Shanghai. Additional file 1: Fig. S14 contextualises the available sub-provincial MMRs of all strain influenza-test positivity within its wider province. Across all provinces, prefectures and counties appear to track the seasonal pattern of their wider province well, with the MMR peaking in the exact same month in 38.6% (95% CI 25.3–52.0%) of provinces, and peaking in the same or ±1 month in 70.8% (95% CI 54.6–87.0%) of provinces. Notably, sub-provincial regions exhibit more extreme values, likely the result of differences in sample size at different spatial scales. A comparison with pre-pandemic MMRs can be seen in Additional file 1: Fig. S9. Overall, similar patterns exist in terms of the latitudinal based annual and semi-annual nature of epidemics across mainland China. Positivity rates, however, appear to be higher in post-pandemic seasons, particularly throughout winter of mid and low-latitude provinces.

Figure 2B highlights the post-pandemic seasonal character of specific influenza strains. Influenza A/H1N1pdm09 and B lineages exhibit strong annual winter peaks, irrespective of latitude, peaking in January and February respectively. A/H3N2 activity varies substantially across China. In high-latitude provinces, A/H3N2 displays a seasonality pattern comparable to both other subtypes, whilst in lower-latitude provinces A/H3N2 activity has a pronounced summer epidemic between June and August. This suggests the semi-annual influenza epidemics experienced by most mid-latitude provinces is not driven by constant activity and resurging epidemics of the same circulating subtypes as in the winter, but rather, a separate summer epidemic of A/H3N2 infections, which is broadly not experienced by higher-latitude provinces. There are, however, a few exceptions to this. Both Beijing and Tianjin experience relatively higher rates of positive A/H3N2 infections in August compared to other provinces in their wider region, corresponding with the summer epidemic in lower-latitude provinces. A similar phenomenon can also be seen in some low-latitude provinces during the winter months (e.g. Shanghai and Guizhou), where A/H3N2 activity increases in line with the epidemic in high-latitude provinces. Although A/H1N1pdm09 and B subtypes exhibit similar seasonality patterns, there are a few distinguishing features. Influenza B epidemics peak on average slightly later and have a more prolonged period of activity, particularly in low-latitude provinces. Whereas, A/H1N1pdm09 has a shorter window of heightened activity where the onset is more consistent across the whole of mainland China.

Although we identified less data on all other influenza outcomes included in this study, particularly in the years prior to the 2009/10 pandemic (Additional file 1: Fig. S5 - S8), we were able to identify some seasonal characteristics in their MMRs (Additional file 1: Fig. S10 - S13). ILI consultation rates appear to exhibit less pronounced seasonal patterns compared to all other outcomes. Mean rates vary in magnitude between administrative regions, suggesting this measure is not uniformly recorded between sites. Proportionally, however, seasonal patterns appear somewhat reflective of the latitudinal patterns observed in influenza test positivity rates. For example, both Liaoning province and Hongqiao county in Tianjin (high-latitude regions) show proportional increases in ILI consultation rates during the winter months, whereas lower-latitude regions (e.g. Fujian) exhibit summer increases in ILI consultations, aligning with increased A/H3N2 positivity in the region. As influenza viruses directly only make up a small fraction of total ILI consultations, less strong seasonal patterns are expected due to activity from other respiratory viruses also circulating throughout the year. In all settings where we identified data on influenza test positive rates among SARI inpatients and influenza-associated excess mortality rates, similar seasonal characters were observed to their general regions MMR of influenza test positivity.

Additional file 1: Fig. S15 shows the linear bivariate relationships between the MMR of each combination of outcomes in each setting where multiple influenza outcomes were identified. All outcomes appear to show positive associations with one other. The overall relationship between mean ILI consultation rates and influenza test positivity rates among ILI outpatients is the weakest we observed, and not statistically significant. However, when examining this relationship in individual administrative regions, stronger positive associations were observed, albeit with substantially varying effect sizes.

### Epidemic onset and duration

**Fig. 3:**
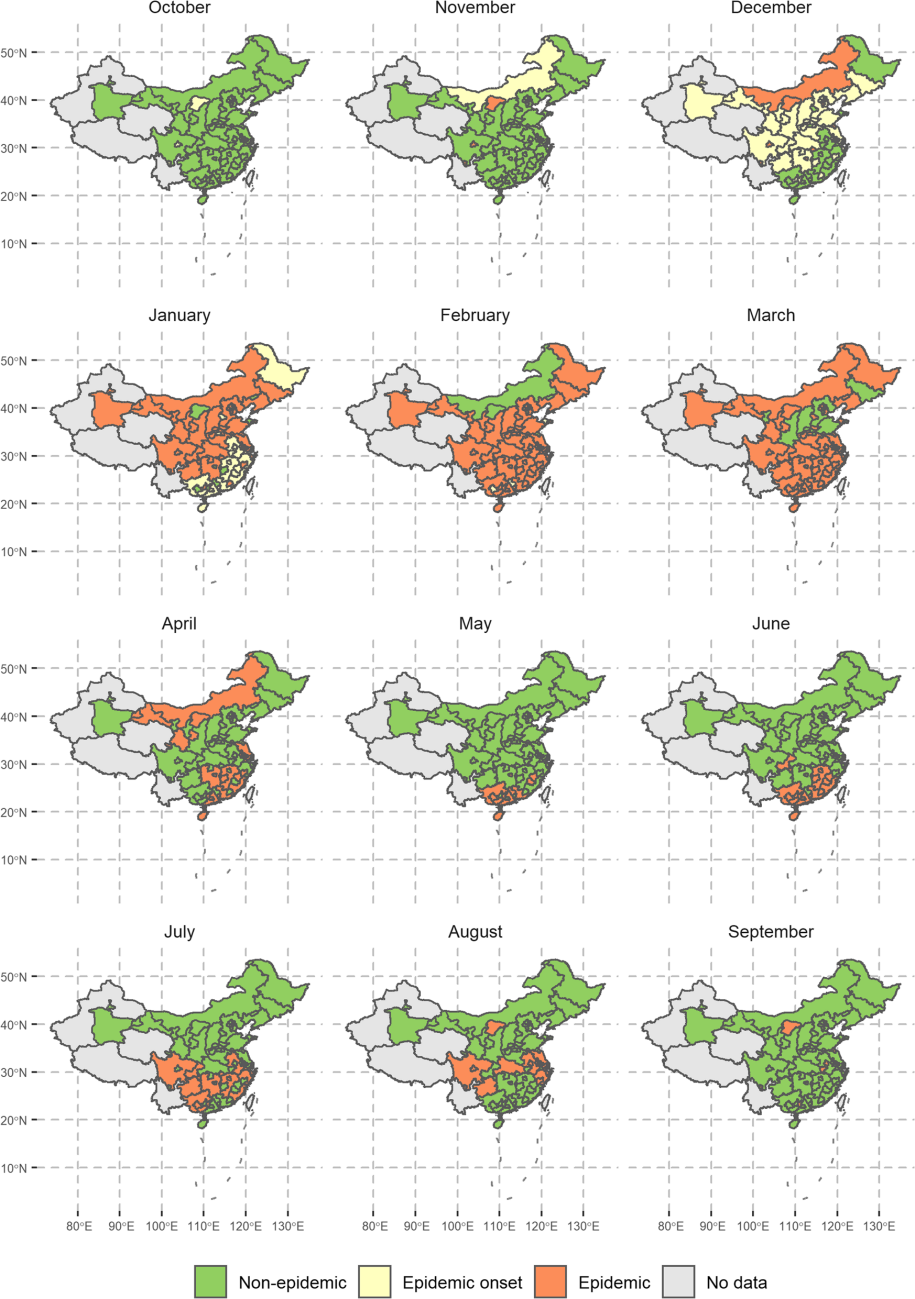
Post-pandemic estimated average epidemic months across mainland China. Based on the MMR of all strain influenza test positivity rates among ILI outpatients. Only displaying administrative regions with available data

Using the post-pandemic MMR of influenza test positivity rate among ILI outpatients, we estimated the average onset month and relative duration of influenza epidemics (Fig. 3). A labelled province-level map is included in Additional file 1: Fig. S20 for reference. Broadly, the onset of influenza epidemics occurs first in Northern China in November. In the following months, epidemics in neighbouring provinces to the south also begin, with the majority of China likely experiencing an epidemic throughout January. During February and March, some northern provinces (e.g. Shanxi and Jilin) begin seeing their epidemics subside. Following this, high and mid-latitude parts of China continuously emerge from their winter epidemics through May, by which point, epidemics are likely only still occurring in southeastern China (e.g. Guangdong, Fujian and Guangxi). From June through August, most regions below 30°N experience a second peak of influenza activity, likely driven by a separate A/H3N2 epidemic. This typically declines to a nationwide low in October and the following epidemiological year’s influenza epidemic is likely once again to occur first in Northern China from November. On average across all provinces, influenza epidemics last approximately 4.74 (95% CI 4.27–5.21) months.

Further maps depicting the estimated average epidemic onset and duration for strain-specific influenza epidemics can be seen in (Additional file 1: Fig. S16 - S18). Each influenza subtype exhibits significantly different average epidemic lengths. A/H3N2 epidemics are on average the longest, lasting approximately 4.83 (95% CI 4.42–5.23) months. This is followed by influenza B and A/H1N1pdm09 with average lengths of 3.57 (95% CI 3.03–4.10) and 2.33 (95% CI 1.95–2.72) months, respectively.

### Epidemic intensity

**Fig. 4:**
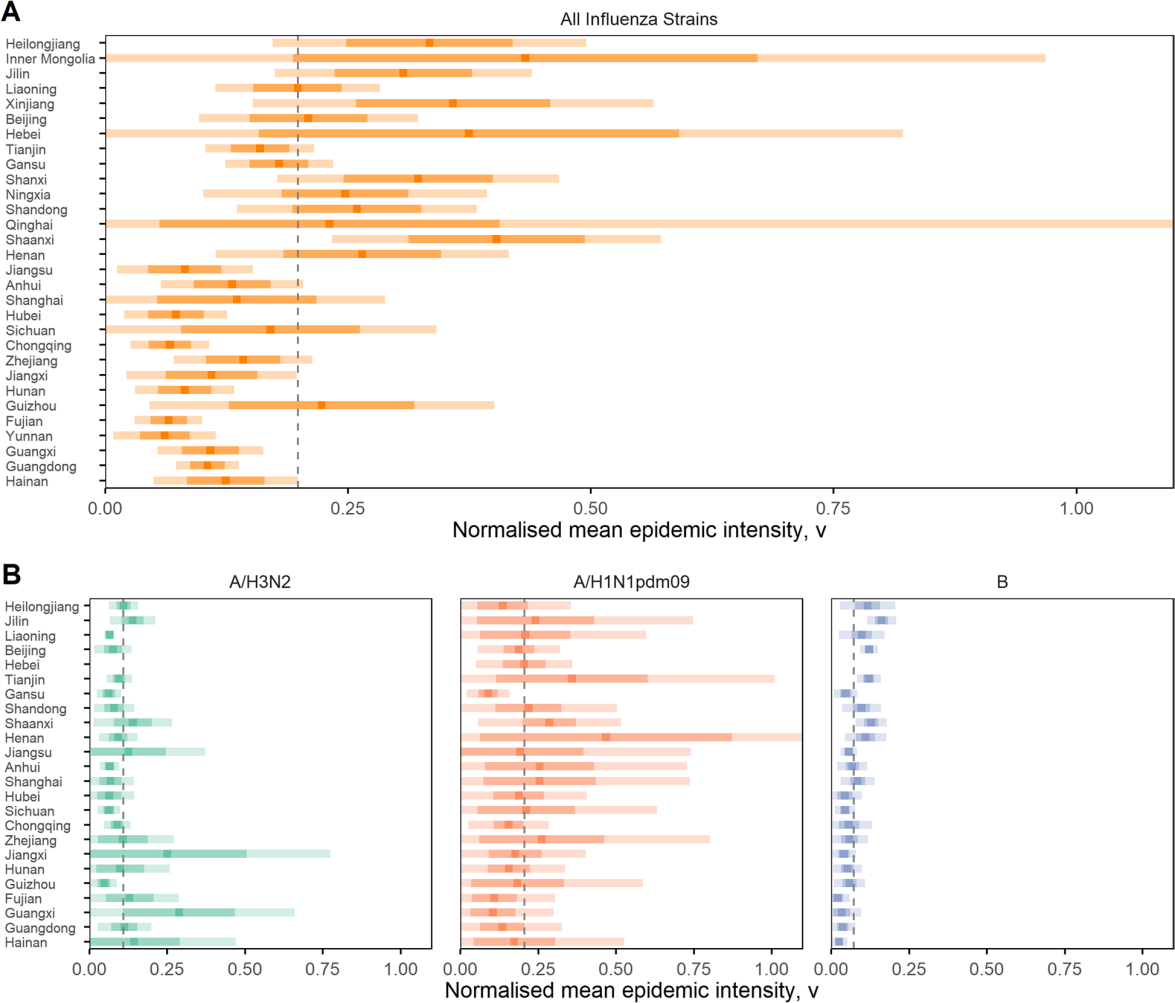
Province-level normalised mean epidemic intensity of influenza test positivity among ILI outpatients, sorted by descending latitude (based on province centroid). **A** Influenza test positivity rate for all common seasonal strains combined. **B** Influenza test positivity rates by A/H3N2, A/H1N1pdm09 and B subtypes. Pandemic period excluded. Centre points denote the province mean intensity; the light and darker shaded bands represent 95% and 75% confidence intervals, respectively. Dashed vertical line represents the group mean

Figure 4 shows the province-level mean relative intensity of influenza epidemics across China, based on test positivity data and sorted by decreasing latitude. In general, higher-latitude provinces experience relatively more intense epidemics than lower-latitude provinces, i.e. they receive a greater proportion of their total annual influenza activity within a smaller proportion of the year. However, intensity in high-latitude provinces appears to be more variable with wide confidence intervals, whilst in mid and low-latitude parts of China epidemics are consistently less intense between epidemiological years. A low mean epidemic intensity also suggests a region may experience semi-annual epidemics, or year-round activity, as influenza activity is more evenly dispersed across a year.

When examining the epidemic intensity of influenza subtypes, the relationship with latitude is more varied (Fig. 4B). Across all provinces, influenza A/H3N2 epidemics have a mean intensity of 0.109 (95% CI 0.083–0.134). This is unsurprising as we have previously shown that A/H3N2 activity is more regularly detected throughout the year as the high and low latitude regions likely experience epidemics at different times, with potential spillover from each resulting in mid-latitude provinces having the least intense epidemics. Broadly, A/H3N2 epidemic intensity is highest in provinces which only experience either a winter or summer epidemic, with the latter showing signs of being relatively more intense (e.g. Jiangxi and Guangxi). The narrow confidence intervals around most high-latitude provinces suggest that they experience a consistent level of intensity between years.

A/H1N1pdm09 epidemics have the highest mean epidemic intensity at 0.206 (95% CI 0.171–0.241), whilst showing no distinct relationships with latitude. This is consistent with our findings that epidemics of this subtype are usually short and only constrained to the winter across all provinces. Uncertainty intervals are high for all provinces, likely due to the smaller sample size of epidemiological years where data was available since its emergence.

Influenza B has the lowest mean relative epidemic intensity of all 3 subtypes studied, with a value of 0.073 (95% CI 0.0563–0.0897). The intensity of influenza B epidemics also follows the same latitudinal pattern as all-strain influenza intensity, however to a lesser extent, with high-latitude provinces exhibiting slightly more intense epidemics than low-latitude provinces.

### Long-term trends

We decomposed province-level influenza test positive rates into their long-term trends, seasonal effects and random components (Additional file 1: Fig. S19). After removing any seasonal influence between 2006 and 2009, there were no clear changes in the trajectory of influenza test-positive rates across mainland China (Additional file 1: Fig. S19B). The occurrence of the 2009/2010 A/H1N1pdm09 pandemic is evident in the long-term trend and random error components as rates sharply increased across mainland China from August 2009 onwards; several months prior to the average onset of the influenza season in northern provinces. Post-pandemic, we find increases in the long-term trend of influenza test positivity rates across all provinces; this suggests that intensified surveillance methods may have increased ascertainment levels. However, an exception to this is the 2012/2013 season which sees a substantial drop in positivity rates, particularly in mid and low-latitude provinces. This is likely due to minimal influenza B activity observed during this season (Additional file 1: Fig. S7). Overall, the seasonal effects observed in each province share strong latitudinal based similarities to those of the MMRs observed in Fig. 2A.

## Discussion

To our knowledge, this is the first systematic review to simultaneously examine the within-year variation of multiple influenza-related health outcomes across mainland China. Utilising a robust search strategy and a range of spatio-temporal analytical methods, we synthesised all the available data to summarise the varied seasonal characteristics experienced across the region. We provide provincial and sub-provincial estimates of monthly average influenza activity, as well as highlighting the likely onset, duration, peak, and intensity of epidemics. In line with other studies, we found seasonal patterns to vary particularly in relation to latitude and geographic location [14–16, 28]. For overall influenza activity, as measured by influenza test positivity among ILI outpatients, we show high-latitude provinces are characterised by having short and intense annual winter epidemics, whilst most mid-latitude and low-latitude provinces experience semi-annual epidemics or year-round activity, respectively. By utilising the most up to date and detailed strain and subtype-specific data available from the literature, we were able to identify varied compositions and timings of influenza epidemics across mainland China, which has important implications for optimising healthcare strategies and immunisation programmes. In addition, by including the use of prefecture and county-level seasonality data in our analysis we were able to show that smaller local areas broadly experience the same general influenza seasonality patterns as their wider province.

Our most notable results are those in relation to the differences observed between seasonal characteristics of specific influenza subtypes across mainland China. In high-latitude provinces, all influenza subtype activity remains largely concentrated in the winter and spring months. This is also true for most mid and low-latitude provinces, with the exception of A/H3N2 epidemics, which likely occur during the summer months. Yu et al., (2013) [14] previously highlighted the semi-annual nature of influenza A epidemics in mid-latitude provinces, however, without stratifying by A/H3N2 and A/H1N1pdm09 subtypes, the specific viral composition behind this seasonal characteristic was not clear. By utilising more recent and strain-specific data, we provide additional information on this phenomenon which has since been observed to broadly occur in southern China as a whole [28], in specific provinces and cities in the region [18], and other subtropical areas outside mainland China [29, 30]. However, evidence from Shu et al., (2010) [15] suggests this viral composition of summer epidemics in southern China may not always be stable and may be subject to changes in the long term, as between 2006 and 2008 summer epidemics were largely driven by A/H1N1 and B/Yamagata activity.

The geographical distinctions we make between the influenza epidemics in high, mid and low-latitude regions are broad categorisations with outliers and a gradient of seasonal patterns in between. For instance, we observe A/H3N2 activity in Beijing and Tianjin to increase slightly during the summer months, aligning with the summer epidemic in mid and low-latitude provinces, and contrasting provinces in their direct proximity. This may in part be due to the high level of travel and immigration these provinces receive, both domestically and internationally [31, 32], resulting in travellers from higher incidence areas (e.g. Southern China and the Southern Hemisphere) potentially seeding chains of transmission outside the traditional main winter influenza epidemics. The reverse relationship can also be observed in the winter months where high positivity rates of A/H3N2 in northern provinces may be leading to a slight increase in infections in Shanghai during December and January. However, the variation in seasonality experienced by these major provinces could be also attributed to several other reasons, such as differences in population age-structure and population density [26, 33].

The disparity in the timing of A/H3N2 epidemics across mainland China may have substantial implications for influenza immunisation programmes. In general, vaccinations in the northern hemisphere are administered throughout the autumn, prior to the winter epidemic; however, this style of roll-out in parts of mainland China may be less effective at reducing incidence in A/H3N2 infections throughout summer epidemics due to waning within-season vaccine effectiveness [34, 35]. Addressing this regional epidemiological nuance may be challenging as repeat revaccination has been shown to be ineffective at enhancing immune response [36, 37]. We further provide evidence that the onset of influenza epidemics tends to be staggered across mainland China. Epidemics occur first in parts of the North of the county and then several months later in the Southeast. Thus, regional-based approaches to vaccination timing may optimise levels of vaccine effectiveness, however, this should be balanced with the practicalities of vaccinating large amounts of the population and the risk of an early influenza season. With the length and intensity of epidemics also varying across the county, regions that experience short epidemics may need to be more adaptable for potential surge capacity compared to regions with a year-round burden of disease.

This study suffers from a number of limitations. Firstly, to enable direct comparisons between the influenza-related seasonal profiles of different administrative regions, we calculated the MMR based on data from multiple seasons. Whilst this method successfully captures the mean seasonal trend across many years, and likely highlights months with regularly increased activity, it may, however, obscure seasonality patterns in settings where activity is variable from year to year. This could result in a situation where the MMR is diluted across more months, potentially giving the impression that a region experiences more prolonged epidemics than it does in reality. However, by using the seasonal decomposition analysis and epidemic intensity to interpret the MMRs, we are able to identify provinces with greater variability in their seasonality patterns. In addition, by separating the MMR aggregation of pre and post-pandemic seasons, and excluding the pandemic period itself (Additional file 1: Fig. S9), we were able to compare if there were any marked differences in the seasonal characteristics. Overall, we observed very similar patterns, suggesting that all-strain influenza test positivity rates among ILI outpatients were not substantially variable between seasons, or, not changing in any particular direction over time. The main difference between pre and post-pandemic seasons we could find was the higher test positivity rates at the peak of epidemics post-pandemic. However, this was not likely a result of any substantial changes in the epidemiology and intensity of influenza epidemics, but rather, the result of improved testing and better ascertainment of cases with the vast expansion of the influenza surveillance network and more widespread use of real-time reverse transcription PCR tests to identify subtypes or lineages [5, 38].

Secondly, as we conducted all of our analysis at the monthly level, our results may suffer from a lack of temporal granularity, as shorter-term changes in activity may be concealed. To standardise the data between studies we aggregated weekly data up to the monthly level, rather than disaggregating down, as this would require making various assumptions about data interpolation. Moreover, a high level of temporal resolution may not be required for the intended purposes of this review. We aimed to provide insights to better inform the planning and optimization of future vaccination programmes and healthcare provision. Therefore, any additional short term fluctuations detected from conducting the analysis at a smaller temporal resolution would likely be the result of noise from the multi-year aggregation of seasonal data, rather than any meaningful seasonal characteristic not captured at the monthly level.

Thirdly, the majority of our analysis relies upon utilising influenza test-positive data among ILI outpatients. This is because the current surveillance systems which record this indicator are well established and widely available across mainland China, and in turn, this data is frequently utilised for epidemiological research. Although this extensive surveillance can provide great insight into the general seasonal characteristics of influenza activity across the region, it does not directly reveal anything about the within-year seasonal variation of disease severity or absolute levels of burden, as it is a function of testing intensity. Whilst we have shown influenza test-positive rates among ILI outpatients to be positively associated with test positivity among SARI inpatients and influenza-associated excess mortality rates in certain settings, this relationship may not be uniform across all of mainland China, as it is only based on a small sample size where both outcomes were available in the same region. Previous studies have estimated the variations in influenza-related mortality across China [5]; however, results are reported at an annual level and do not examine the within-year variation. This information may be particularly useful for planning healthcare provision and anticipating surge capacity at the peak of epidemics. Greater surveillance of these measures is therefore required to provide further insight into the within-year seasonal disparities experienced across China.

Fourth, due to many studies included in this review not disaggregating influenza B outcomes by B/Yamagata and B/Victoria lineages, we decided to conduct our seasonal analysis for influenza B as a whole. This reduced our ability to distinguish any seasonal differences between these lineages. However, doing so would be based on sparse data and would not be geographically representative of mainland China. Previous studies suggest B/Yamagata and B/Victoria annually alternate in dominance of total influenza B activity [18]. This observation may have important implications for the planning of vaccination strategies and the decision to roll out quadrivalent or trivalent vaccines to prevent mismatches with circulation [39]. This observation may have important implications for the planning of vaccination strategies and the decision to roll out quadrivalent or trivalent vaccines to prevent mismatches with circulation. However, since the emergence of SARS-CoV-2 in late 2019, a broad range of interventions have been enacted by governments worldwide to reduce its transmission. As a result, this has led to a substantial reduction in the transmission of many other respiratory viruses, with very few cases of influenza B/Yamagata being reported globally in 2020 [40]. Current evidence suggests the lineage may have been eliminated, as there are no known sustained animal reservoirs, or evidence of animal to human transmission [41]. Further surveillance over the coming years is required to better understand this phenomenon, as it may have considerable implications in deciding future components of influenza vaccines.

Finally, our use of digitised data in the analysis may be subject to some slight inaccuracies. However, we believe that acquiring a much larger and richer dataset through the use of this method than otherwise would have been possible by just relying on traditional data requests, or just utalising the raw data provided by some studies, substantially outweighs the potential of slight inaccuracies in the data. With this extraction method, we were able to include data encompassing a much larger proportion of mainland China, at multiple spatial scales, for multiple influenza-associated health outcomes, over a greater time horizon, allowing for a more complete synthesis and review of regional-based within-year variations of influenza activity across mainland China.

Future work should utilise the data collected and reviewed here to inform influenza-associated burden of disease estimates at multiple spatial and temporal scales across mainland China. Further, vaccine impact analyses should account for these spatio-temporal variations to inform future vaccination policy decisions.

## Conclusion

By synthesising all the available data from the literature, this review and analysis has strengthened the understanding of the geographic and within-year variations of seasonal influenza activity across mainland China, at multiple levels of disease severity. In addition to reaffirming the current knowledge base, by using the most up to date data we notably identified the viral composition of seasonal epidemics, along with their likely onset and peak, to vary by broad latitudinal and geographic regions, suggesting the need for regional specific healthcare strategies and immunisation programmes to reduce that burden of disease across the country.

## Supplementary Information

**Additional file 1: Appendix 1**. Database search terms. **Appendix 2**. Inclusion and exclusion criteria. **Appendix 3**. Data cleaning and formatting. **Appendix 4**. Calculating mean monthly rate. **Appendix 5**. Calculating epidemic duration and onset. **Appendix 6**. Calculating epidemic intensity. **Appendix 7**. Calculating long-term trends. **Appendix 8**. Additional figures and tables. **Figure S1**. Estimating Beijing province monthly all strain influenza test positivity rate amongst ILI outpatients time-series from multiple eligible studies. **Figure S2**. Number of studies included in quantitative synthesis by publication year and language. **Figure S3**. Hierarchical map displaying the number of studies identified reporting suitable time-series data in each administrative region for each influenza health outcome. **Figure S4**. Comparison between distribution of administrative regions represented by review and mainland China overall, by A) GDP per capita (USD) & B) Total population. **Figure S5**. Monthly ILI consultation rate among all outpatients, grouped and sorted by province-level latitude. **Figure S6**. Monthly all-strain influenza test positivity rate amongst ILI outpatients, grouped and sorted by province-level latitude. **Figure S7**. Monthly strain specific influenza test positivity rates amongst ILI outpatients, grouped and sorted by province-level latitude. **Figure S8**. Monthly rates of influenza associated health outcomes. A) Influenza test positivity rate among SARI inpatients. B) Influenza-associated excess mortality rate among respiratory mortality (per 100 000 person-years). C) Influenza-associated excess mortality rate among all-cause mortality (per 100 000 person-years). **Figure S9**. Comparison of province-level pre and post 2009/10 influenza pandemic mean monthly rate (MMR) of all influenza strain test positivity, among ILI outpatient consultations. **Figure S10**. Mean monthly rate (MMR) of ILI consultation rate among all outpatient consultations. Only post 09/10 pandemic years included. **Figure S11**. Mean monthly rate (MMR) of all influenza strain test positivity, among ILI outpatient consultations. Only post 09/10 pandemic years included. **Figure S12**. Mean monthly rate (MMR) of strain specific influenza test positivity, among ILI outpatient consultations. Only post 09/10 pandemic years included. **Figure S13**. Mean monthly rate (MMR) of multiple influenza associated health outcomes. A) Influenza test positivity rate among SARI inpatients. B) Influenza-associated excess mortality rate among respiratory mortality (per 100 000 person-years). C) Influenza-associated excess mortality rate among all-cause mortality (per 100 000 person-years). **Figure S14**. Provincial and sub-provincial administrative regions mean monthly rates (MMR) of all-strain influenza test positivity among ILI outpatients, arranged as an approximate geographical representation of mainland China. **Figure S15**. Mean monthly rate (MMR) cross outcome comparison. **Figure S16**. Influenza A/H3N2 estimated average epidemic months across mainland China. **Figure S17**. Influenza A/H1N1pdm09 estimated average epidemic months across mainland China. **Figure S18**. Influenza B estimated average epidemic months across mainland China. **Figure S19**. Seasonal decomposition of province-level all-strain influenza test positivity among ILI outpatients. **Figure S20**. Province-level map of China. **Table S1**. Final studies included in quantitative synthesis after title, abstract and full text screening.

## Data Availability

All relevant data and materials used in this analysis can be found in the manuscript, additional file and the accompanying GitHub repository: https://github.com/EIDECDIA/China_influenza_seasonality

## Abbreviations

95% CI: 95% confidence interval
ILI: Influenza-like illness
MMR: Mean monthly rate
NIP: National immunisation programme
PRISMA: Preferred Reporting Items for Systematic Reviews and Meta-Analyses
SARI: Severe acute respiratory infection
