# Supplementary material for: Regional-based within-year seasonal variations in influenza-related health outcomes across mainland China: a systematic review and spatio-temporal analysis
Source: BMC Med. 2022 Feb 10;20:58. doi: 10.1186/s12916-022-02269-5 (PMC8830135; doi:10.1186/s12916-022-02269-5)
Supplement: Supplementary file 1 — Additional file 1: Appendix 1. Database search terms. Appendix 2. Inclusion and exclusion criteria. Appendix 3. Data cleaning and formatting. Appendix 4. Calculating mean monthly rate. Appendix 5. Calculating epidemic duration and onset. Appendix 6. Calculating epidemic intensity. Appendix 7. Calculating long-term trends. Appendix 8. Additional figures and tables. Figure S1. Estimating Beijing province monthly all strain influenza test positivity rate amongst ILI outpatients time-series from multiple eligible studies. Figure S2. Number of studies included in quantitative synthesis by publication year and language. Figure S3. Hierarchical map displaying the number of studies identified reporting suitable time-series data in each administrative region for each influenza health outcome. Figure S4. Comparison between distribution of administrative regions represented by review and mainland China overall, by A) GDP per capita (USD) & B) Total population. Figure S5. Monthly ILI consultation rate among all outpatients, grouped and sorted by province-level latitude. Figure S6. Monthly all-strain influenza test positivity rate amongst ILI outpatients, grouped and sorted by province-level latitude. Figure S7. Monthly strain specific influenza test positivity rates amongst ILI outpatients, grouped and sorted by province-level latitude. Figure S8. Monthly rates of influenza associated health outcomes. A) Influenza test positivity rate among SARI inpatients. B) Influenza-associated excess mortality rate among respiratory mortality (per 100 000 person-years). C) Influenza-associated excess mortality rate among all-cause mortality (per 100 000 person-years). Figure S9. Comparison of province-level pre and post 2009/10 influenza pandemic mean monthly rate (MMR) of all influenza strain test positivity, among ILI outpatient consultations. Figure S10. Mean monthly rate (MMR) of ILI consultation rate among all outpatient consultations. Only post 09/10 pandemic years included. Figure S1 [file 12916_2022_2269_MOESM1_ESM.docx]

### **Appendix**

##### **1. Database search terms**

We adapted our set of search terms to be most effective for the nuances of different database searching mechanisms. This can be seen as follows:

###### Web of science -

ALL=(Influenza OR flu OR influenza-like illness OR ILI)

AND

ALL=(burden OR incidence OR prevalence OR mortality OR death OR morbidity OR outpatient OR hospitalization OR admission OR hospitalized OR inpatient OR virology OR Virologic OR virological OR “positive rates” OR “lab confirmed”)

AND

ALL=(China OR Anhui OR Beijing OR Chongqing OR Fujian OR Gansu OR Guangdong OR Guangxi OR Guizhou OR Hainan OR Hebei OR Heilongjiang OR Henan OR Hubei OR Hunan OR Neimenggu OR Inner Mongolia OR Jiangsu OR Jiangxi OR Jilin OR Liaoning OR Ningxia OR Qinghai OR Shaanxi OR Shandong OR Shanghai OR Shanxi OR Sichuan OR Tianjin OR Xinjiang OR Yunnan OR Zhejiang OR Tibet OR Xizang)

AND

All=(time series OR timeseries OR time-series OR longitudinal OR multiple seasons OR seasonality OR seasonal OR weekly OR monthly OR temporal)

###### PubMed terms -

(((("influenza, human"[MeSH Terms] OR ("influenza"[All Fields] AND "human"[All Fields]) OR "human influenza"[All Fields] OR "influenza"[All Fields]) OR ("influenza, human"[MeSH Terms] OR ("influenza"[All Fields] AND "human"[All Fields]) OR "human influenza"[All Fields] OR "flu"[All Fields]) OR influenza-like illness[all] OR ILI[all]) AND (burden[all] OR ("epidemiology"[Subheading] OR "epidemiology"[All Fields] OR "incidence"[All Fields] OR "incidence"[MeSH Terms]) OR ("epidemiology"[Subheading] OR "epidemiology"[All Fields] OR "prevalence"[All Fields] OR "prevalence"[MeSH Terms]) OR ("mortality"[Subheading] OR "mortality"[All Fields] OR "mortality"[MeSH Terms]) OR ("death"[MeSH Terms] OR "death"[All Fields]) OR ("epidemiology"[Subheading] OR "epidemiology"[All Fields] OR "morbidity"[All Fields] OR "morbidity"[MeSH Terms]) OR ("outpatients"[MeSH Terms] OR "outpatients"[All Fields] OR "outpatient"[All Fields]) OR ("hospitalisation"[All Fields] OR "hospitalization"[MeSH Terms] OR "hospitalization"[All Fields]) OR admission[all] OR hospitalized[all] OR ("inpatients"[MeSH Terms] OR "inpatients"[All Fields] OR "inpatient"[All Fields]) OR ("virology"[Subheading] OR "virology"[All Fields] OR "virology"[MeSH Terms]) OR ("virology"[MeSH Terms] OR "virology"[All Fields] OR "virologic"[All Fields]) OR ("virology"[MeSH Terms] OR "virology"[All Fields] OR "virological"[All Fields]) OR "positive rates"[all] OR "lab confirmed"[all])) AND (("china"[MeSH Terms] OR "china"[All Fields]) OR Anhui[all] OR ("beijing"[MeSH Terms] OR "beijing"[All Fields]) OR Chongqing[all] OR Fujian[all] OR Gansu[all] OR Guangdong[all] OR Guangxi[all] OR Guizhou[all] OR Hainan[all] OR Hebei[all] OR Heilongjiang[all] OR Henan[all] OR Hubei[all] OR Hunan[all] OR ("china"[MeSH Terms] OR "china"[All Fields] OR ("inner"[All Fields] AND "mongolia"[All Fields]) OR "inner mongolia"[All Fields]) OR Jiangsu[all] OR Jiangxi[all] OR Jilin[all] OR Liaoning[all] OR Ningxia[all] OR Qinghai[all] OR Shaanxi[all] OR Shandong[all] OR Shanghai[all] OR Shanxi[all] OR Sichuan[all] OR Tianjin[all] OR Xinjiang[all] OR Yunnan[all] OR Zhejiang[all])) AND (time series[all] OR timeseries[all] OR time-series[all] OR longitudinal[all] OR multiple seasons[all] OR seasonality[all] OR seasonal[All Fields] OR weekly[all] OR monthly[all] OR temporal[all]) AND (English[lang] OR Chinese[lang]))

###### Wanfang -

(主题="流感"+"流行性感冒"+"甲流"+"乙流") AND (摘要="流行病学"+"疾病负担"+"发病率"+"现患率"+"患病率"+"死亡率"+"死亡数"+"门诊"+"住院"+"入院"+"病毒学"+"阳性率"+"实验室确诊"+"实验室诊断") AND (摘要="时间序列"+"时序图"+"时序分析"+"纵向"+"多季节"+"跨季节"+"季节性"+"实时监控"+"周统计"+"月统计")

###### CNKI -

(SU='流感' OR SU='流行性感冒' OR SU='甲流' OR SU='乙流') AND (AB='流行病学' OR AB='疾病负担' OR AB='发病率' OR AB= '现患率' OR AB= '患病率' OR AB='死亡率' OR AB='死亡数' OR AB='门诊' OR AB='住院' OR AB='入院' OR AB='病毒学' OR AB='阳性率' OR AB='实验室确诊' OR AB='实验室诊断') AND (AB='时间序列' OR AB='时序图' OR AB='时序分析' OR AB='纵向' OR AB='多季节' OR AB='跨季节' OR AB='季节性' OR AB='实时监控' OR AB='周统计'OR AB='月统计')

##### **2. Inclusion and exclusion criteria**

###### Inclusion criteria

We included peer-reviewed articles in English and Chinese languages, published between January 1, 2000, to September 31, 2019. Eligible articles were those reporting whole population based quantitative time-series data on any of the four following influenza outcomes:

1. ILI consultation rates (among all outpatient consultations)
2. Influenza test positive rates among ILI outpatients
   1. A/H3H2 test positive rate among ILI outpatients
   2. A/H1N1pdm09 test positive rate among ILI outpatients
   3. Influenza B test positive rate among ILI outpatients
3. Influenza test positive rates among SARI inpatients
4. Influenza associated excess mortality

Any other influenza associated outcomes or metrics were not considered by this study. This includes outcomes which are calculated from combinations of our 4 selected outcomes e.g. ILI consultation rates multiplied by influenza test positive rates (ILI+). Further, as pre-pandemic A/H1N1 currently contributes little to the overall influenza-associated burden of disease, this outcome was not included in the additional subtype analysis (points 2a – 2c). In addition, studies must report data at either the province, prefecture or county-level of administrative regions. Data on specific subpopulations were not included in the analysis (e.g. age-group or profession-specific) as a lack of consistent categorizations were used in different settings across China, thus making it difficult to compare rates between subpopulations.

###### Exclusion criteria

Studies were excluded from the review for if they:

1. Were not about natural human infection with influenza
2. Did not report time-series data on any of the pre-defined desired influenza outcomes (see inclusion criteria)
3. Only reported time-series data at a weekly or monthly temporal resolution
4. Examined a study period of less than 1 year
5. Only reported non-date specific time-series data, such as only reporting average multi-year rates
6. Only reported time-series data on patients with comorbidities, such as influenza surveillance among individuals with chronic medical conditions.
7. Were a systematic review or meta analysis without any new primary data
8. Only reported time-series data at the national level, or, data outside mainland China (this includes China’s special administrative regions and Taiwan)
9. Not available to access freely.
10. Only reported time-series data in visualizations from which data was unable to accurately extracted data from, or visualizations of poor image quality

##### **3. Data cleaning and formatting**

After extracting data from eligible studies, we aggregated values from each distinct time-series to the monthly level. To achieve this, data points reported at the weekly level were grouped into their respective month, based upon which month the final day of the week fell (e.g. Sunday). We then calculated the group mean to create a new monthly value and time-series. No changes were made to datasets which were originally reported at the monthly level.

From the monthly data, we constructed a unique time-series for each influenza health outcome, in each administrative region where data was available. In many cases, this involved joining data from multiple separate studies describing the exact same outcome in the exact same administrative region. Furthermore, in some cases monthly data points were temporally overlapping, thus, to ensure consistency when combining overlapping data points we took the following procedure:

1. If only minor discrepancies were observed between overlapping data points we calculated the mean, as this was likely the result of slight inaccuracies arising from the digitisation extraction process.
2. If large divergences between overlapping points were observed we re-examined the studies to diagnose the underlying reason. Differences could arise for a number of reasons, for instance differences in sample populations. However, if a study appeared to be reporting inaccurate data (e.g. if the data was outdated and then revised in a more recent study), it was removed from the review.
3. If neither studies’ data appeared to be inaccurate, we calculated the mean of the given data points.

Figure S1 demonstrates this process for influenza test positive rates among ILI outpatients in Beijing province.

*
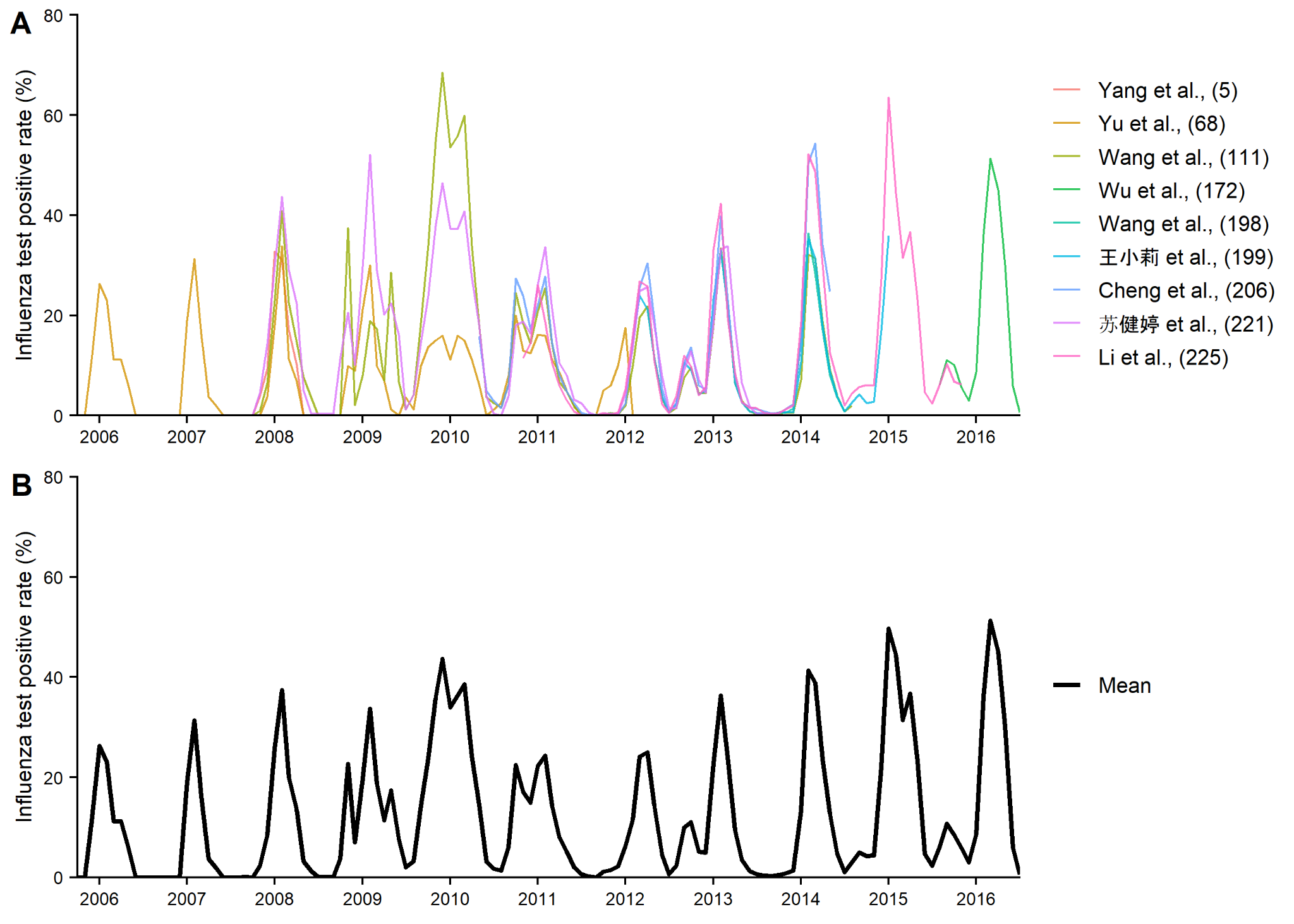
*

*Figure S1: Estimating Beijing province monthly all strain influenza test positivity rate amongst ILI outpatients time-series from multiple eligible studies. A) Time-series data from separate studies, B) Reconstructed time-series. Numbers in parentheses correspond to the study reference number in Table S2.*

In contrast to ILI consultation rates and influenza test positivity rates, influenza associated excess mortality is typically reported as a rate per 100 000 person-years, as opposed to a percentage or proportion. Thus, when aggregating weekly estimates to the monthly level we had to take additional steps. We calculated the total weekly excess death case numbers based upon the weekly rate and the total population. Using the same weekly to monthly grouping method described previously, we calculated the total number of deaths for each month and then converted this back into a rate per 100 000 person-years.

Figure S5 - S8 shows the reconstructed time-series for all outcomes in each administrative region where eligible data was available.

##### **4. Calculating mean monthly rate**

We estimated the MMR of a given influenza indicator, in any administrative region, by the following formula:

$${MMR}_{i} = \frac{\sum_{i=1}^{n} R_{i}}{n_{i}}$$

Where *i* represents the month of the year, *R* denotes the influenza rate at month *i*, and *n* signifies the number of years of data available at month *i*. We calculated the MMR separately for both pre and post 2009/10 influenza pandemic periods, whilst excluding the impact of pandemic period itself. In addition, we only calculated the MMR in settings with greater than 2 years of monthly observations.

##### **5. Calculating epidemic duration and onset**

Adapted from a method described in Li et al (2019)[11], we estimated the average relative duration of an influenza epidemic in a given administrative region based upon the proportion of the total average annual influenza activity (MMR) which occurred in each month of the year. In each region, we sorted months in descending order from highest to lowest annual MMR percentage and then calculated the rolling cumulative sum. The minimum number of months needed to cross the relative epidemic threshold of 75% of total annual MMR activity were defined as *“Epidemic”* months, whilst the remainder were denoted *“Non-epidemic”* months. The frequency of *“Epidemic”* months thus denotes the average epidemic duration. This method allows for multiple peaks within a year as it does not require the *“Epidemic”* months to be consecutive. The first *“Epidemic”* month following the start of the influenza epidemiological year, defined here as October, was classed as the *“Epidemic onset”* month. This aligns the seasonality patterns more broadly with other influenza seasons in the northern hemisphere.

##### **6. Calculating epidemic intensity**

The epidemic intensity of a given influenza indicator, in a given epidemiological year, *v_j_*, can be summarised by the following equation:

$$v_{j}= (-\sum_{i} p_{ij} log p_{ij})^{-1}$$

Where in any region, *p_ij_* represents the proportion of annual influenza activity that occurred in month *i*, of year *j*. We normalised values of *v_j_* between 0 and 1 by subtracting the global minimum, and then dividing by the global maximum minus the global minimum. This enables direct comparisons between epidemics in each region, and across years in relative terms. For specific influenza subtypes we normalised epidemic intensity across all lineages to enable comparisons between epidemics of different influenza subtypes. We further calculated the normalised mean epidemic intensity in regions with individual estimates across multiple epidemiological years.

Province-level estimates of epidemic intensity were calculated using data from our reconstructed time-series of influenza test-positivity among ILI outpatients (Figure S6). We defined influenza epidemiological years as starting in October. Calculating the epidemic intensity of a given epidemiological year requires complete data for each month of that year. Therefore we excluded years from the analysis with any missing monthly data. We also excluded epidemiological years in a given region where influenza was observed in fewer than 3 months of the year, as this was likely an indication of incomplete surveillance and reporting rates in that year, rather than a highly intense epidemic. Further, whilst estimating the epidemic intensity of strain and subtype-specific influenza epidemics we also excluded any epidemiological years where a nationwide epidemic for a specific strain did not occur, e.g. A/H1N1pdm09 in the 2015-2016 season.

##### **7. Calculating long-term trends**

Using a classical additive seasonal decomposition model we decomposed our province-level time-series of influenza test positivity among ILI outpatients into their respective trend, seasonality and random error components. The overall model can be summarised as:

$$R_{i}=M_{i}+S_{i}+E_{i}$$

Where in a given province *i* represents the month, *R* denotes the test positivity rate at month *i,* *M* signifies the trend, *S* describes the seasonal effect, and *E* shows the remaining random error.

The trend at month *i* (*M_i_*) is calculated as a 12 month moving average based on the lag and lead of the observed rate:

$$M_{i}=\frac{\frac{1}{2}R_{i-6}+R_{i-5}+R_{i-4}+R_{i-3}+R_{i-2}+R_{i-1}+R_{i}+R_{i+1}+R_{i+2}+R_{i+3}+R_{i+4}+R_{i+5}+{\frac{1}{2}R}_{i+6}}{12}$$

Thus, subtracting the trend (*M_i_*) from the observed time-series (*R_i_)* leaves the combined seasonal effect and random error. To isolate the overall seasonal effect, we calculate the monthly mean of the remaining seasonal estimates and random error. This average seasonal monthly sequence is then repeated over all years of available data to provide estimates of *S_i_*. Finally, the random error (*E_i_)* is estimated by subtracting the trend and seasonal effect from the observed time-series:

$$E_{i}=R_{i}-M_{i}-S_{i}$$

#####

##### **8. Additional figures and tables**


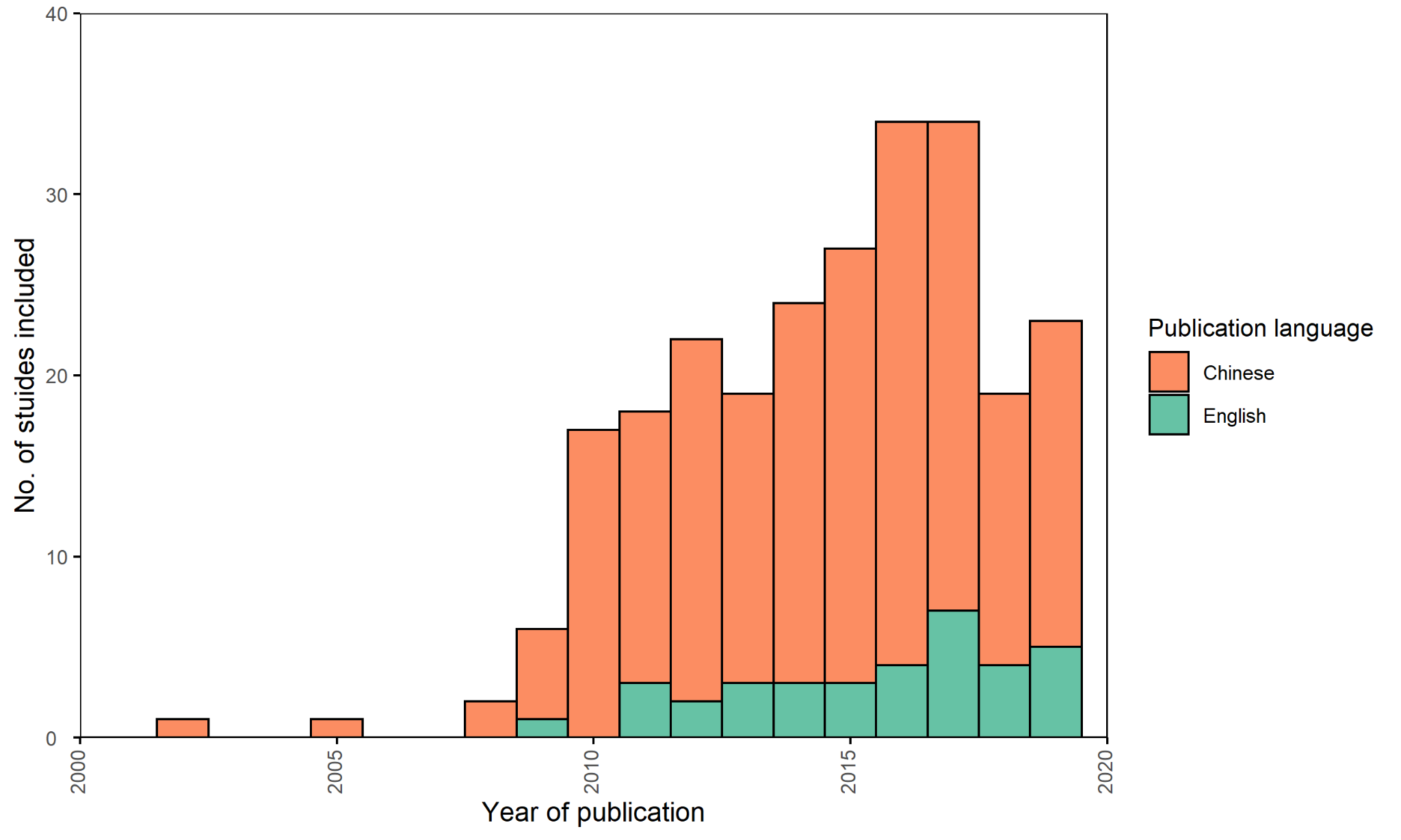


*Figure S2: Number of studies included in quantitative synthesis by publication year and language.*


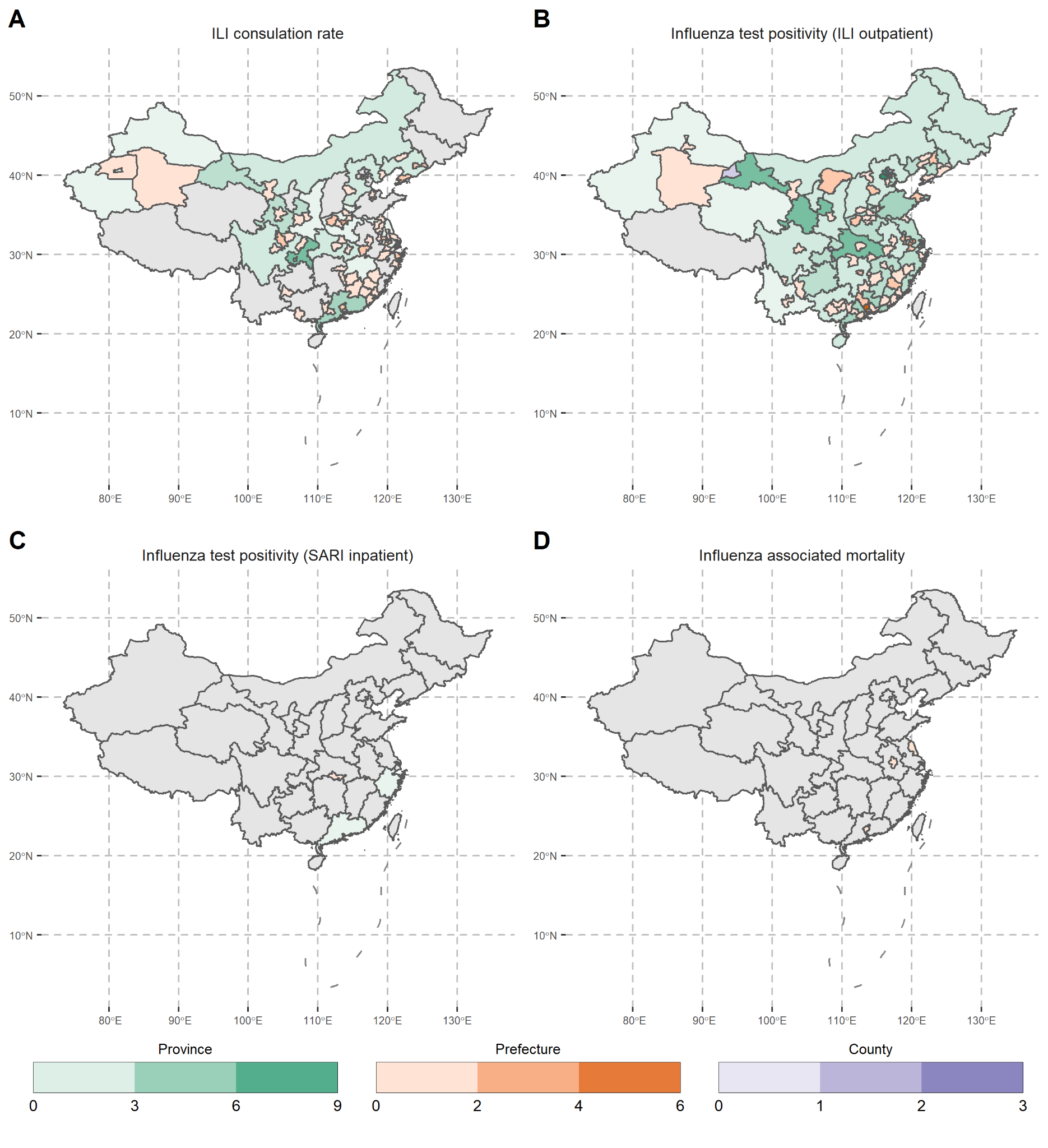


*Figure S3: Hierarchical map displaying the number of studies identified reporting suitable time-series data in each administrative region for each influenza health outcome. A) ILI consultation rate (outpatients), B) Influenza test positivity rate (ILI outpatients), C) Influenza test positivity rate (SARI inpatients) & D) Influenza associated excess mortality rate. Background displays all provinces and only prefecture and county-level administrative regions reporting data are layered on top.*

*
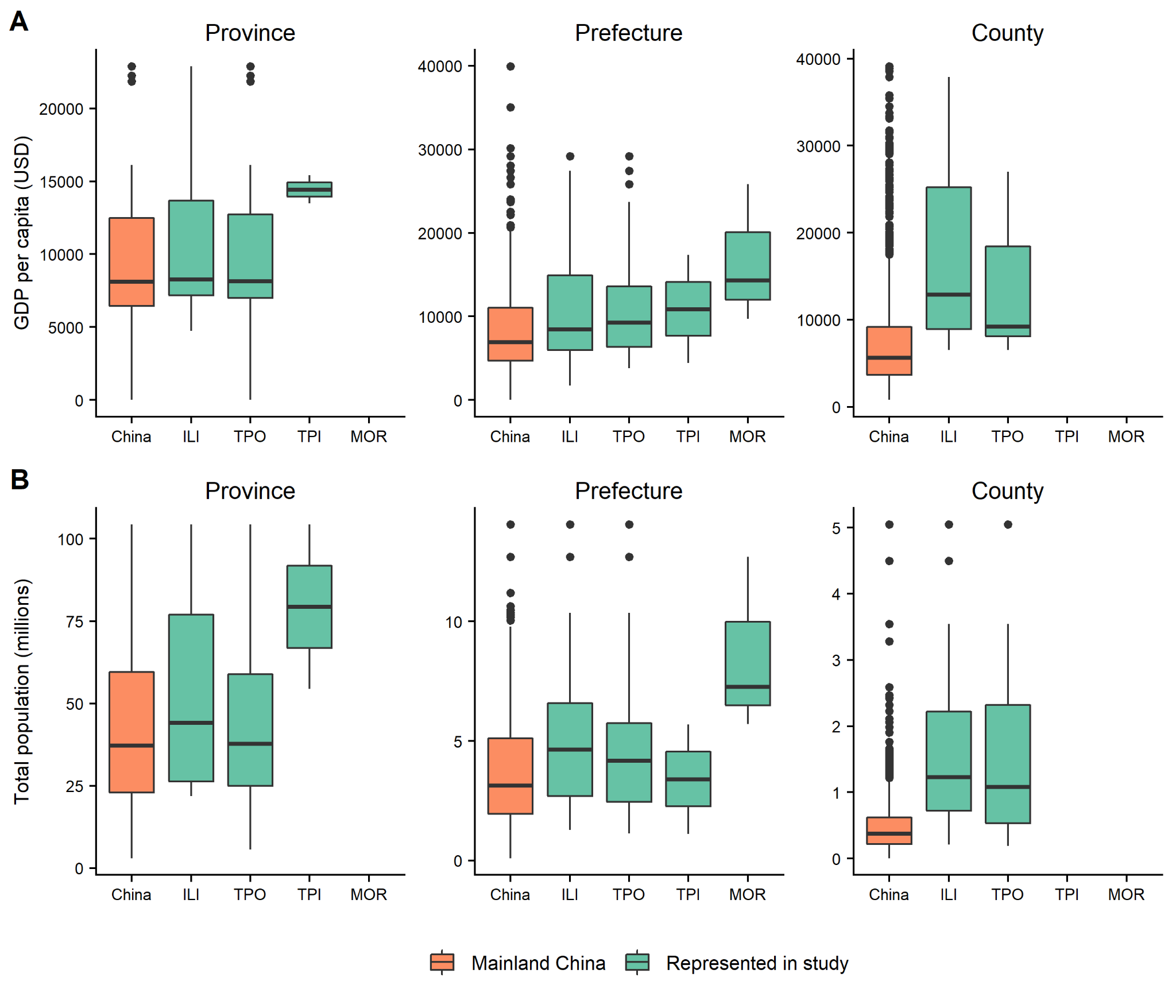
*

*Figure S4: Comparison between distribution of administrative regions represented by review and mainland China overall, by A) GDP per capita (USD) & B) Total population. Based on 2010 census data. ILI = ILI consultation rate, TPO = Influenza test positivity rate among ILI outpatients, TPI = Influenza test positivity rates among SARI inpatients, MOR = Influenza associated excess mortality rate per 100 000 person years. GDP per capita data cropped at 40 000 USD.*

*
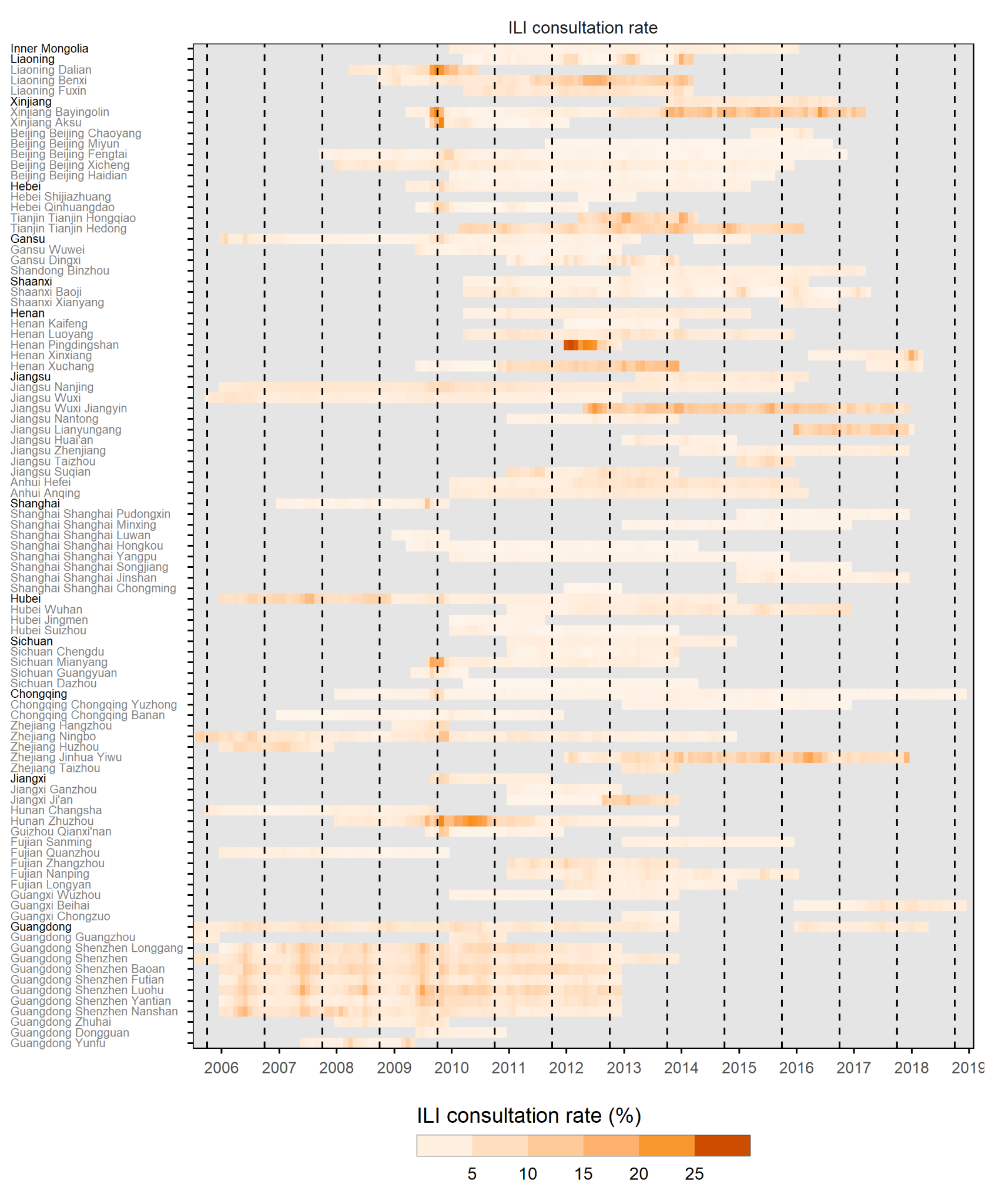
*

*Figure S5: Monthly ILI consultation rate among all outpatients, grouped and sorted by province-level latitude. Black y-axis text denotes provinces, grey text signifies prefecture or county-level administrative regions. Dashed lines indicate October 1st (start of a new influenza epidemiological year). Grey months represent no data.*

*
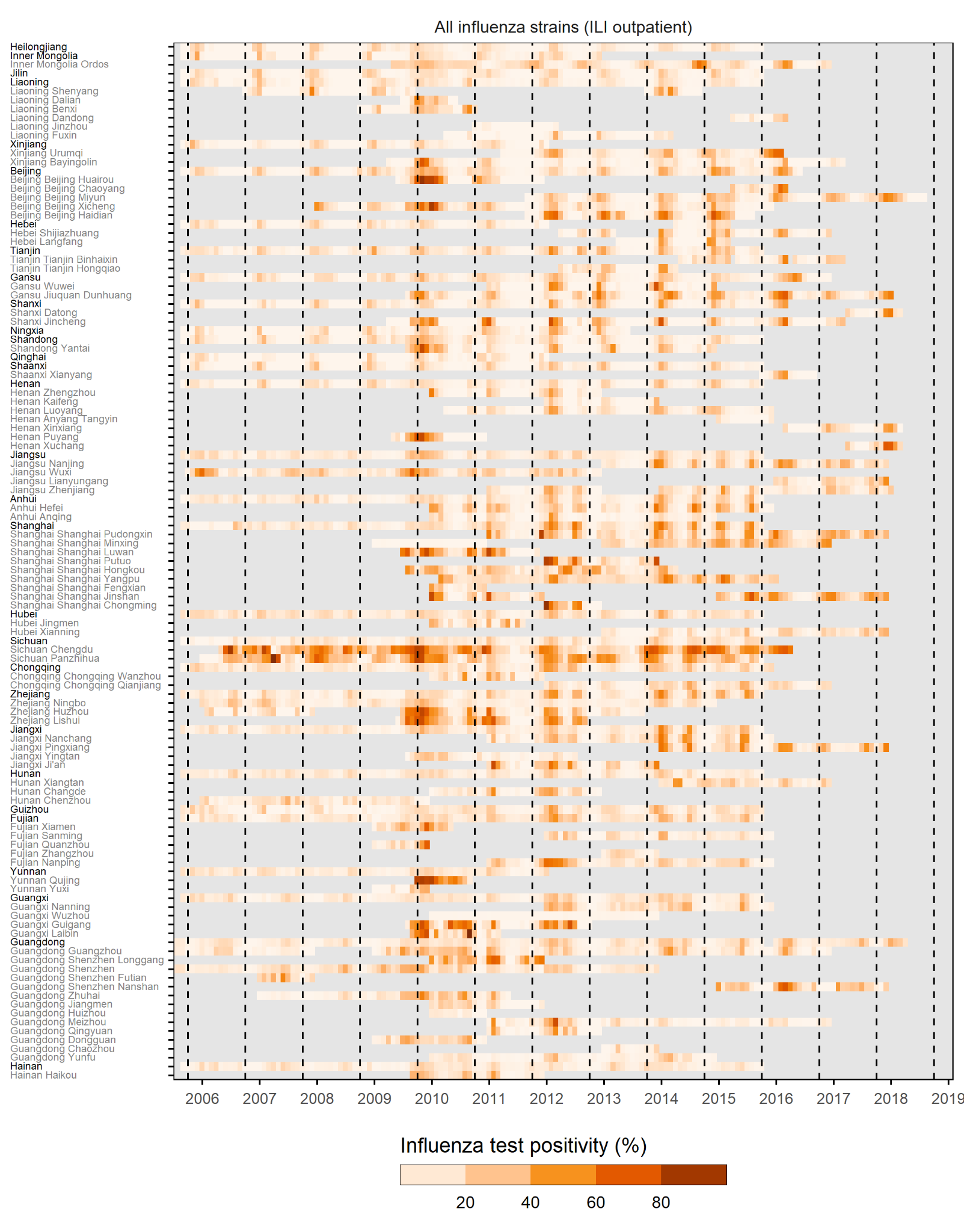
*

*Figure S6: Monthly all-strain influenza test positivity rate amongst ILI outpatients, grouped and sorted by province-level latitude. Black y-axis text denotes provinces, grey text signifies prefecture or county-level administrative regions. Dashed lines indicate October 1st (start of a new influenza epidemiological year). Grey months denote no data.*

*
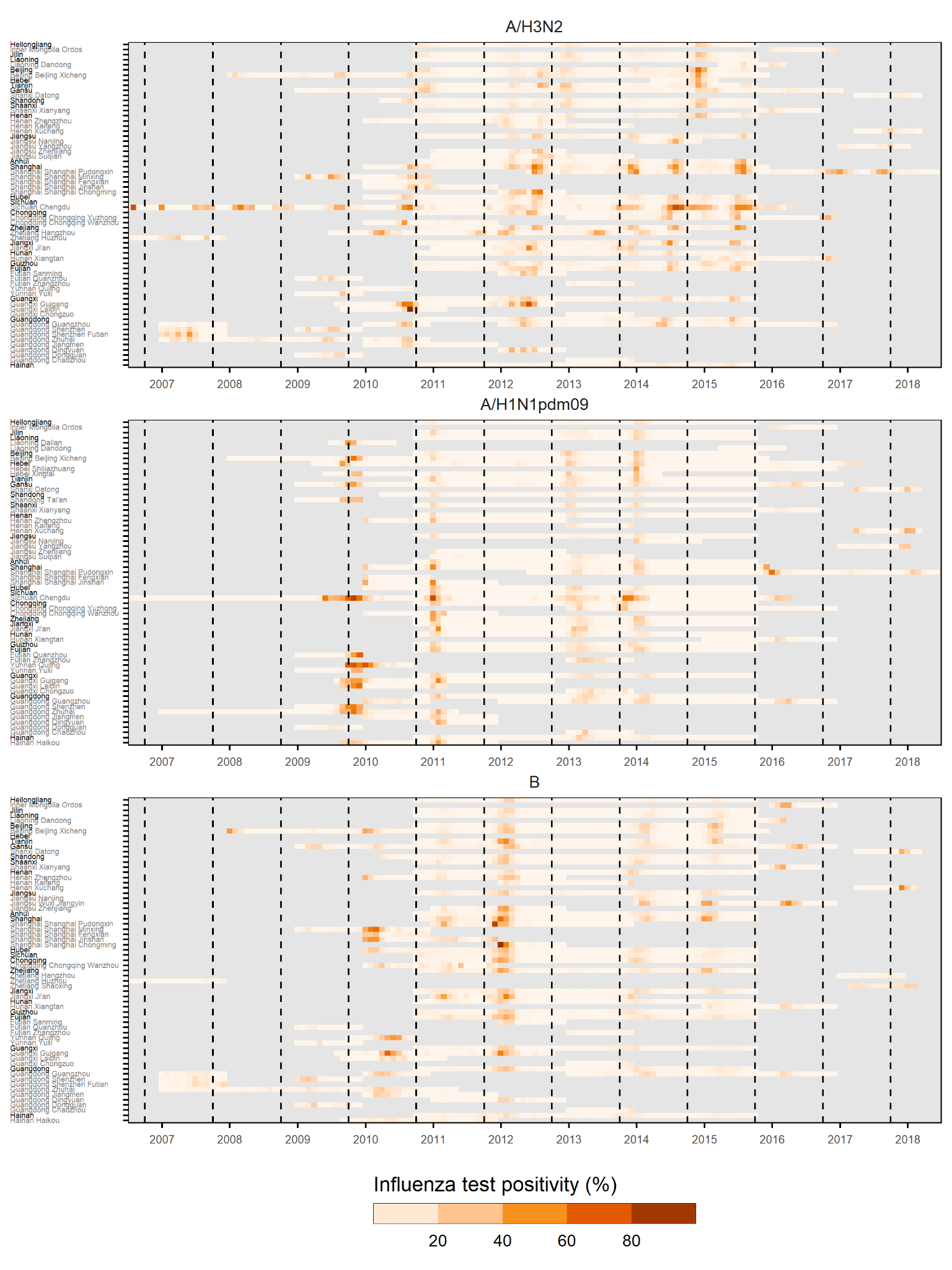
*

*Figure S7: Monthly strain specific influenza test positivity rates amongst ILI outpatients, grouped and sorted by province-level latitude. Black y-axis text denotes provinces, grey text signifies prefecture or county-level administrative regions. Dashed lines indicate October 1st (start of a new influenza epidemiological year). Grey months denote no data.*

*
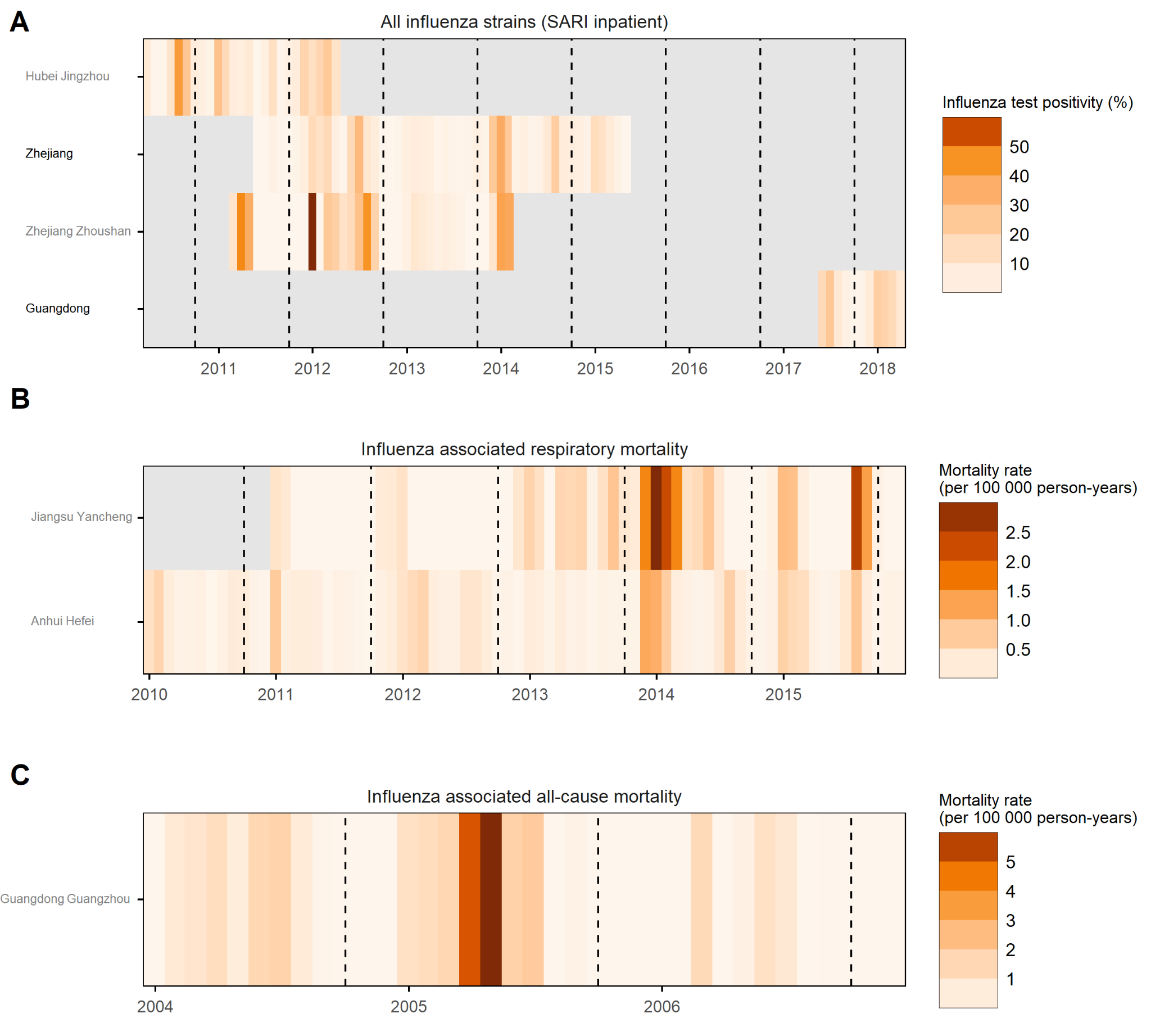
*

*Figure S8: Monthly rates of influenza associated health outcomes. A) Influenza test positivity rate among SARI inpatients. B) Influenza-associated excess mortality rate among respiratory mortality (per 100 000 person-years). C) Influenza-associated excess mortality rate among all-cause mortality (per 100 000 person-years). Regions are sorted and grouped by descending province latitude. Black y-axis text denotes provinces, grey text signifies prefecture or county-level administrative regions. Grey months denote no data. Dashed lines indicate October 1st (start of a new influenza epidemiological year).*


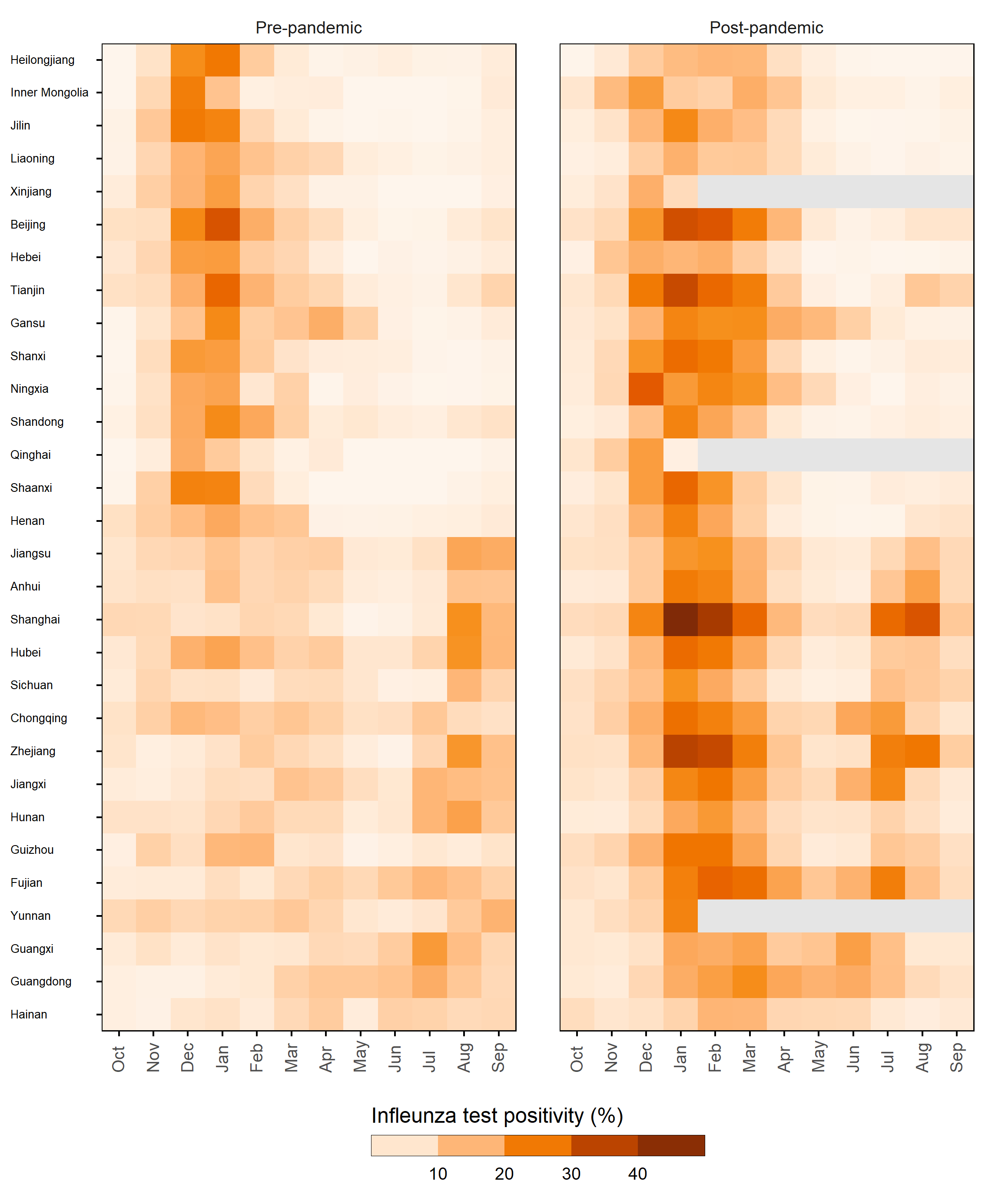


*Figure S9: Comparison of province-level pre and post 2009/10 influenza pandemic mean monthly rate (MMR) of all influenza strain test positivity, among ILI outpatient consultations. Pandemic period itself is excluded. Regions are sorted and grouped by descending province latitude.*


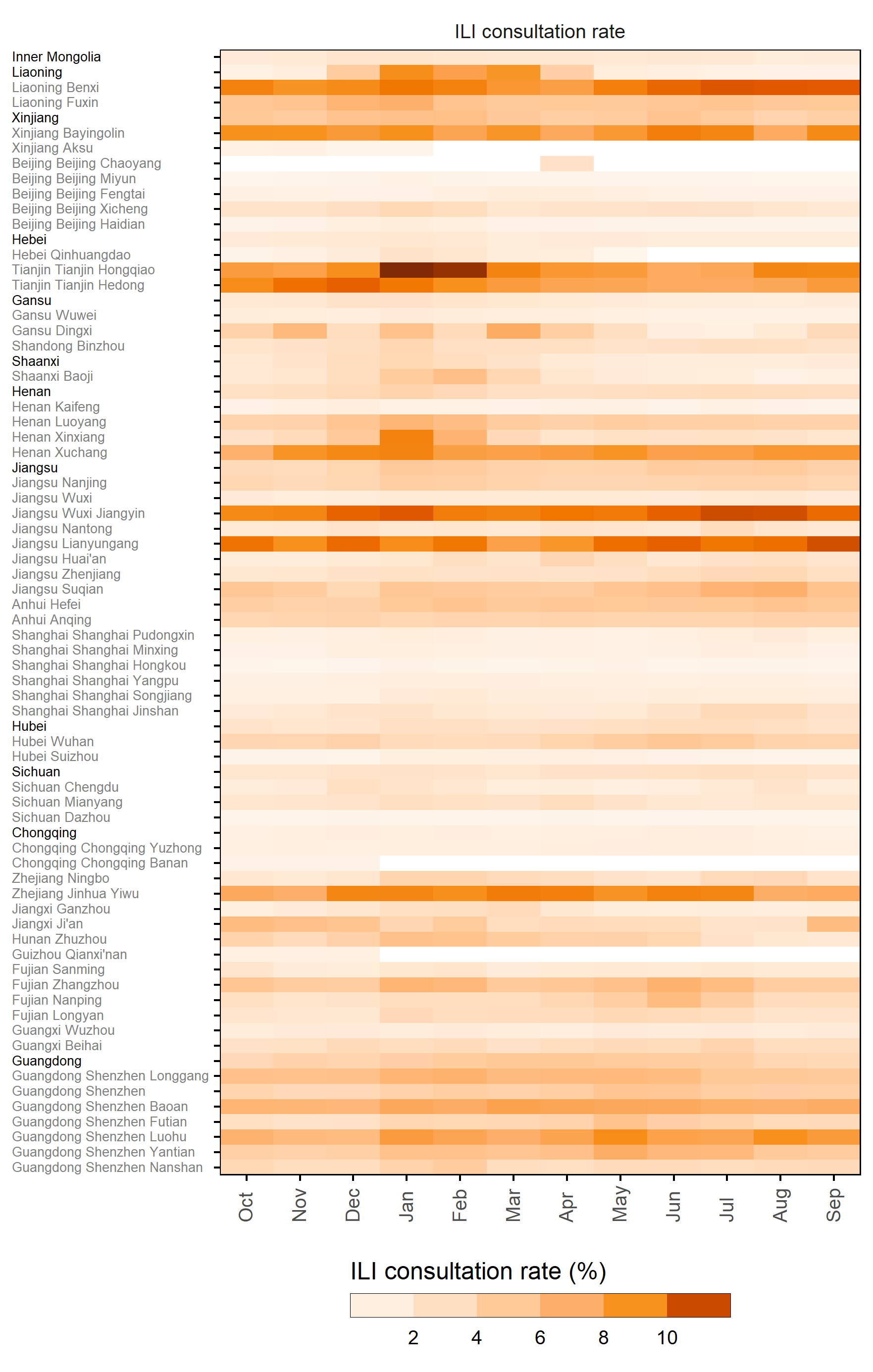


*Figure S10: Mean monthly rate (MMR) of ILI consultation rate among all outpatient consultations. Only post 09/10 pandemic years included. Regions are sorted and grouped by descending province latitude. Black y-axis text denotes provinces, grey text signifies prefecture or county-level administrative regions.*


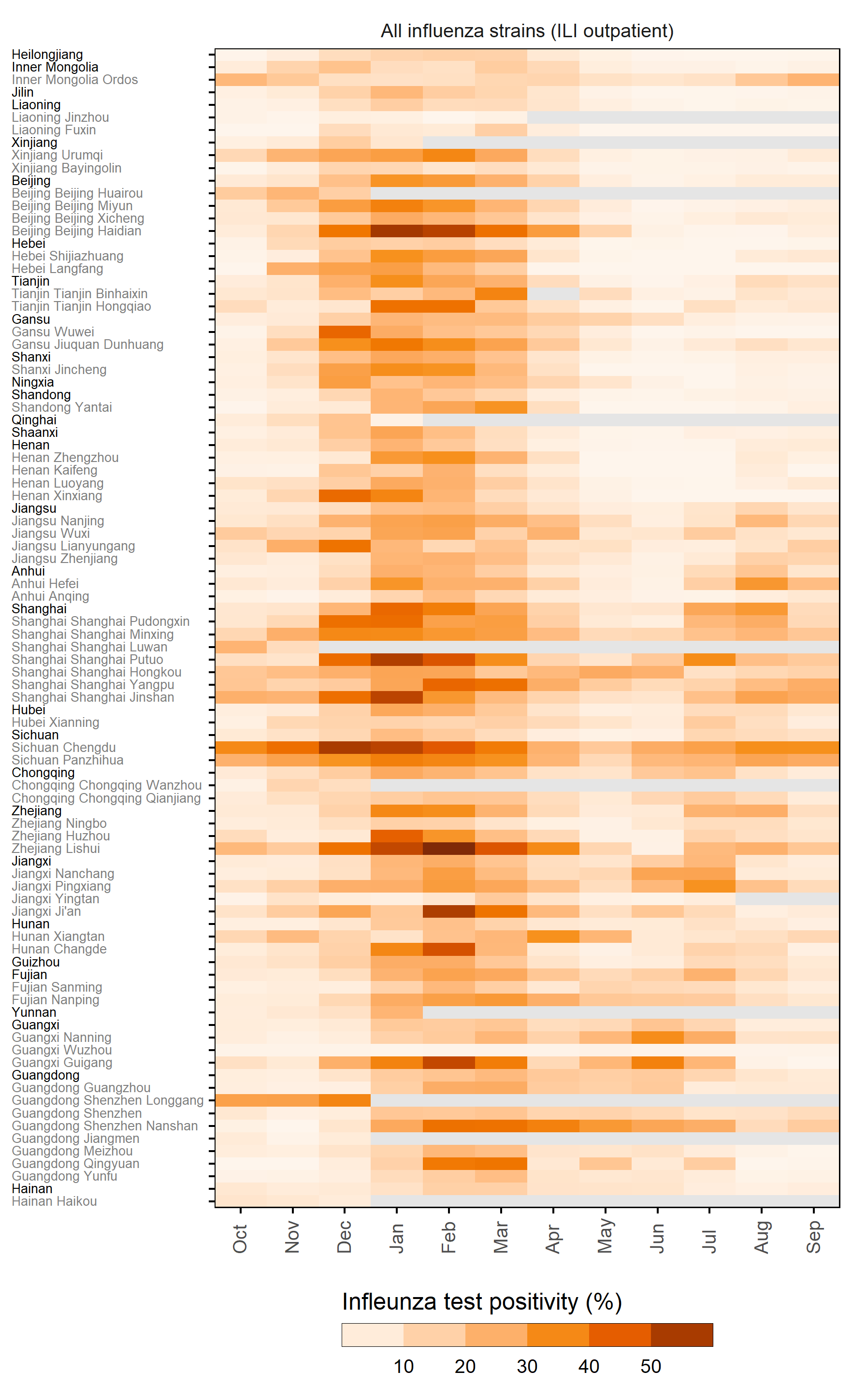


*Figure S11: Mean monthly rate (MMR) of all influenza strain test positivity, among ILI outpatient consultations. Only post 09/10 pandemic years included. Regions are sorted and grouped by descending province latitude. Black y-axis text denotes provinces, grey text signifies prefecture or county-level administrative regions.*


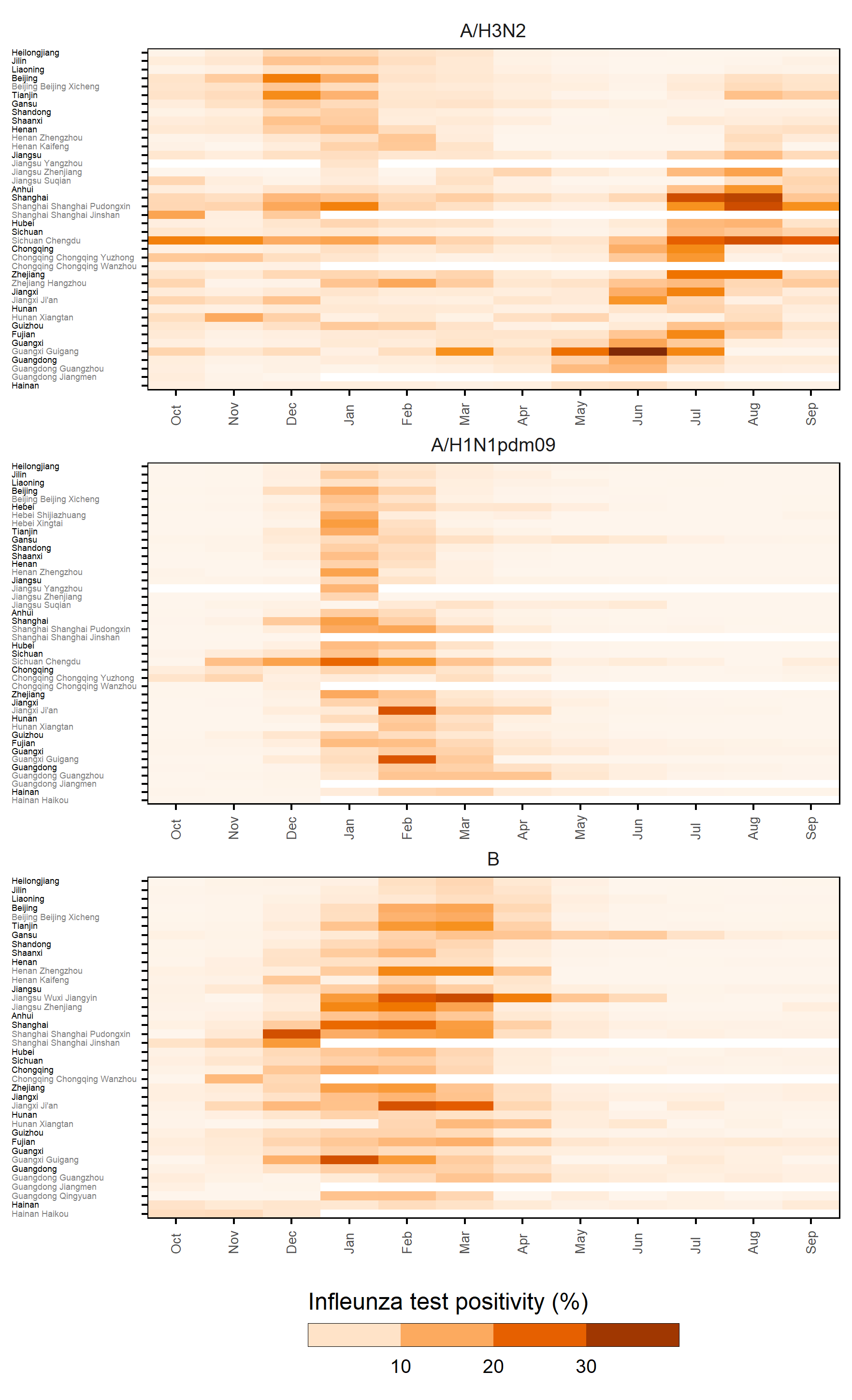


*Figure S12: Mean monthly rate (MMR) of strain specific influenza test positivity, among ILI outpatient consultations. Only post 09/10 pandemic years included. Regions are sorted and grouped by descending province latitude. Black y-axis text denotes provinces, grey text signifies prefecture or county-level administrative regions.*


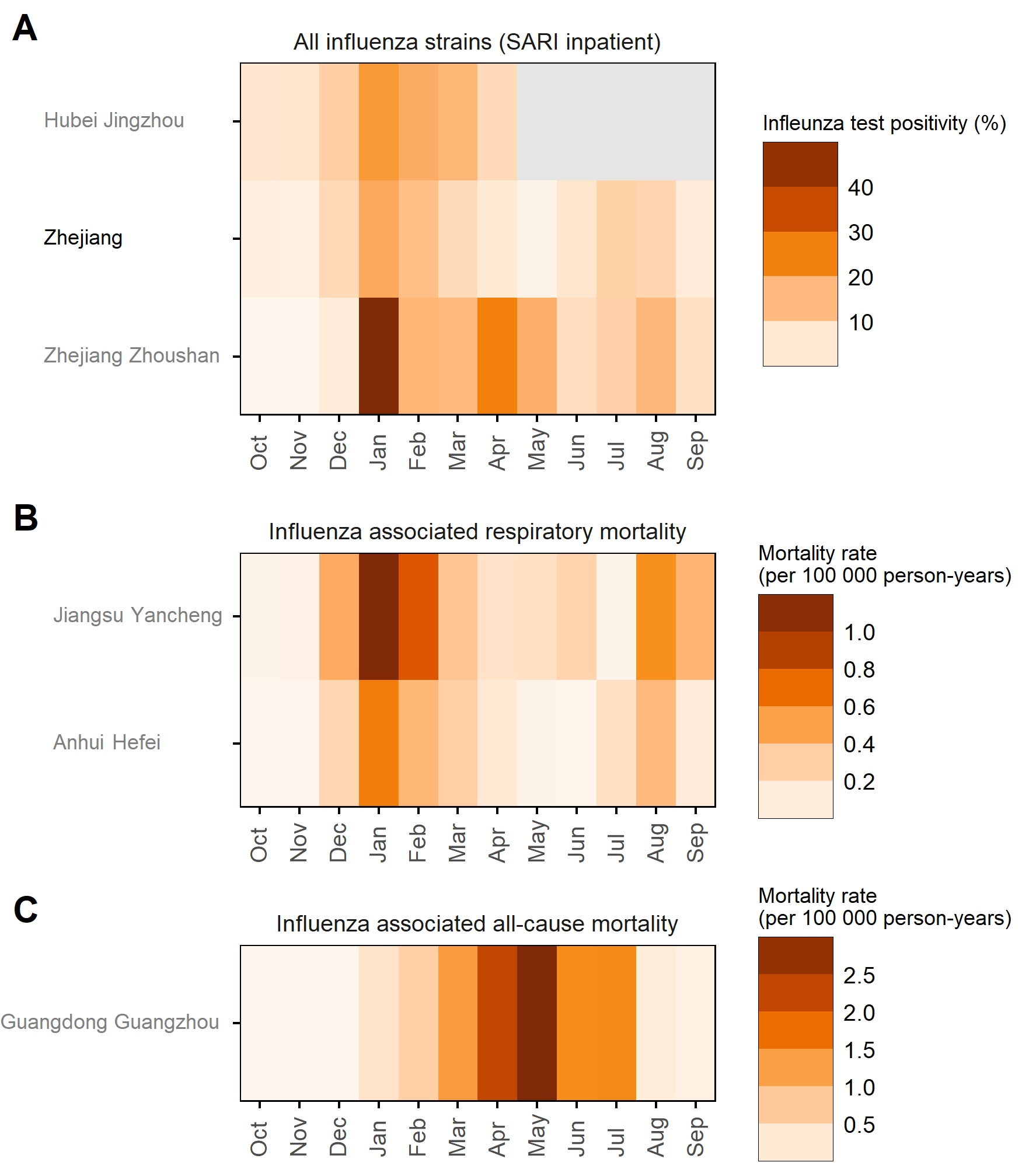


*Figure S13: Mean monthly rate (MMR) of multiple influenza associated health outcomes. A) Influenza test positivity rate among SARI inpatients. B) Influenza-associated excess mortality rate among respiratory mortality (per 100 000 person-years). C) Influenza-associated excess mortality rate among all-cause mortality (per 100 000 person-years). Regions are sorted and grouped by descending province latitude. Black y-axis text denotes provinces, grey text signifies prefecture or county-level administrative regions.*


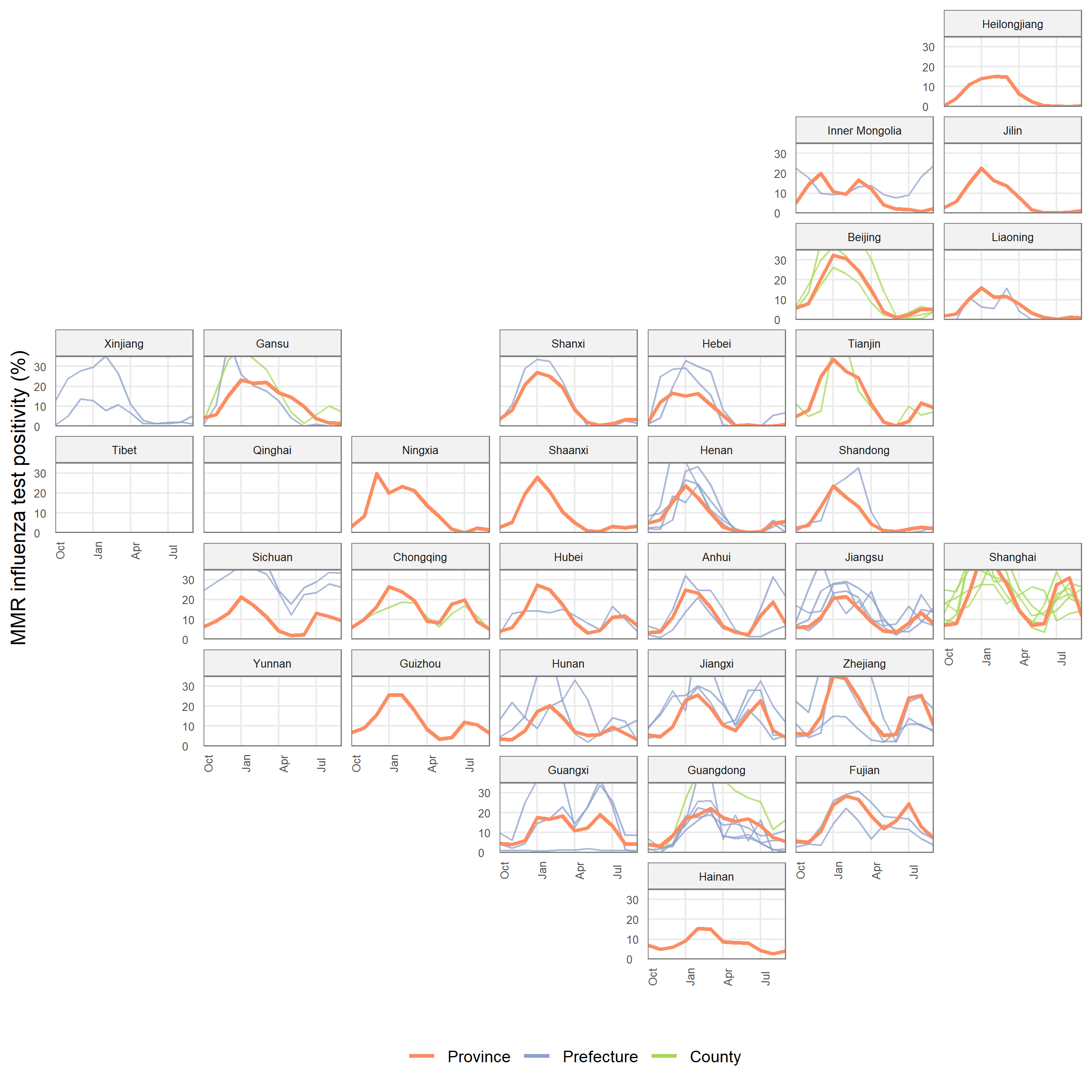


*Figure S14: Provincial and sub-provincial administrative regions mean monthly rates (MMR) of all-strain influenza test positivity among ILI outpatients, arranged as an approximate geographical representation of mainland China.*

*
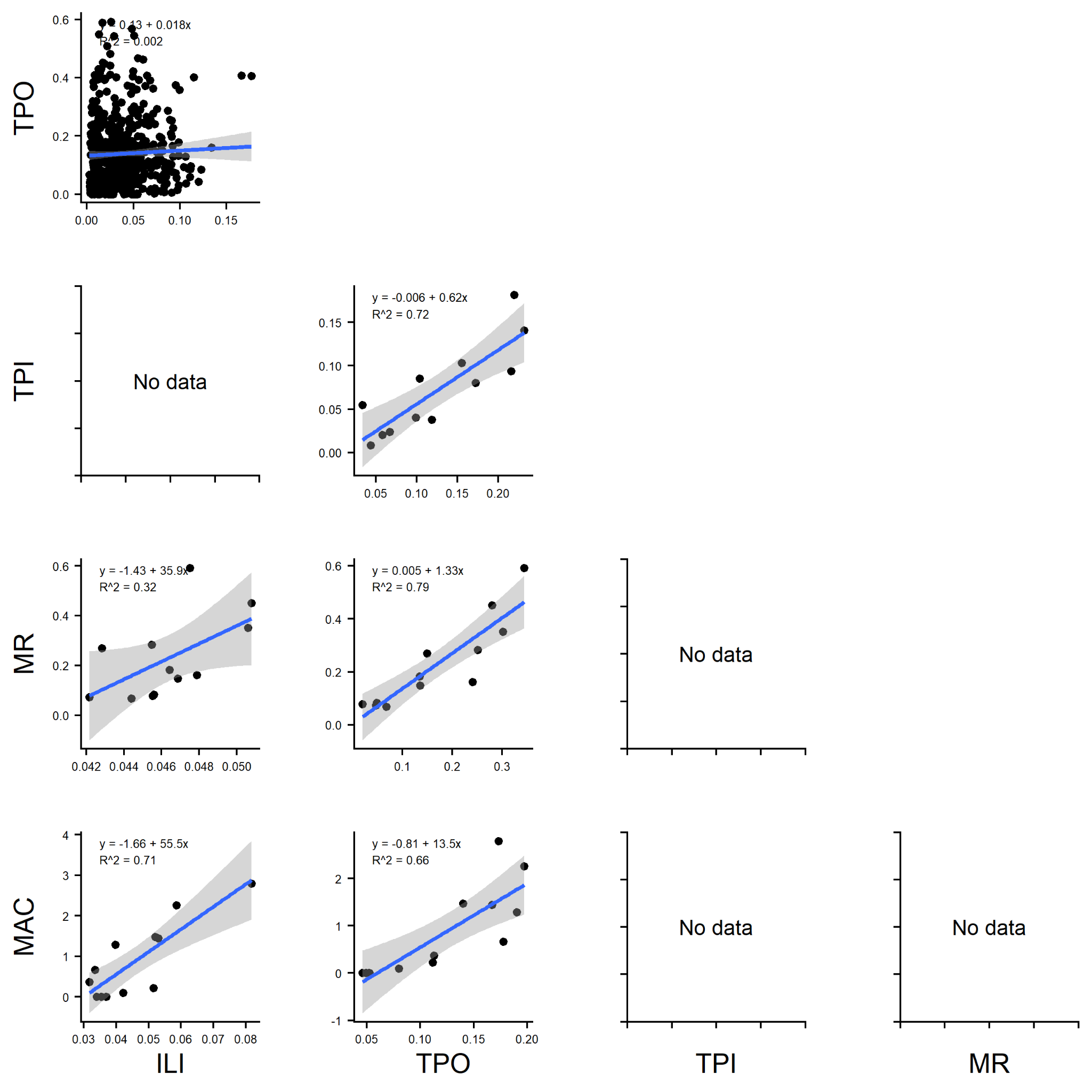
*

*Figure S15: Mean monthly rate (MMR) cross outcome comparison. Points represent the MMR of a given influenza outcome in a given month and administrative region. ILI = ILI consultation rate, TPO = Influenza test positivity rate among ILI outpatients, TPI = Influenza test positivity rates among SARI inpatients, MR = Influenza associated excess mortality rate per 100 000 person years among respiratory mortality & MAC = Influenza associated excess mortality rate per 100 000 person years among all-cause mortality.*


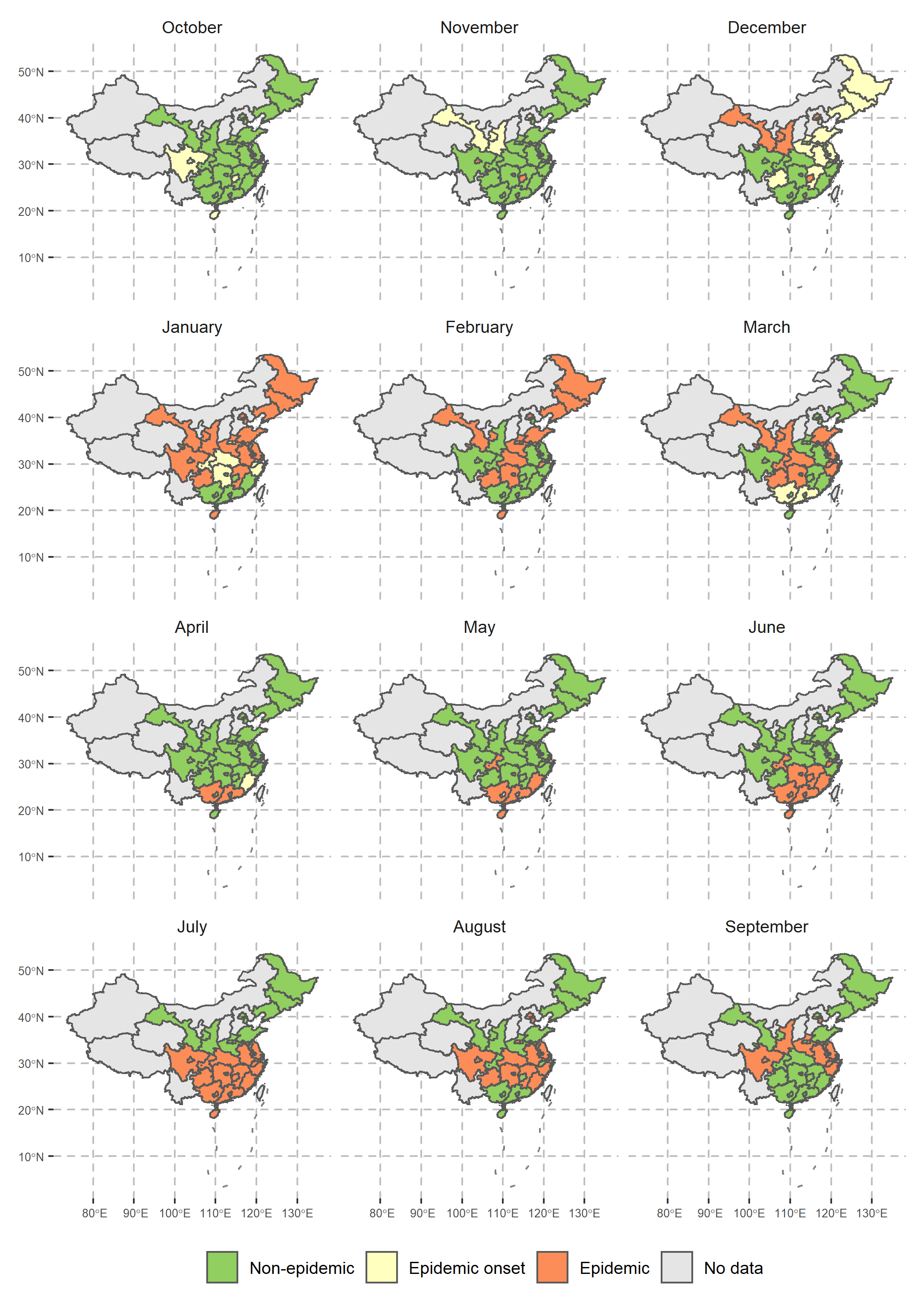


*Figure S16: Influenza A/H3N2 estimated average epidemic months across mainland China. Based on the MMR of influenza test positivity rates among ILI outpatients. Only displaying administrative regions with available data.*


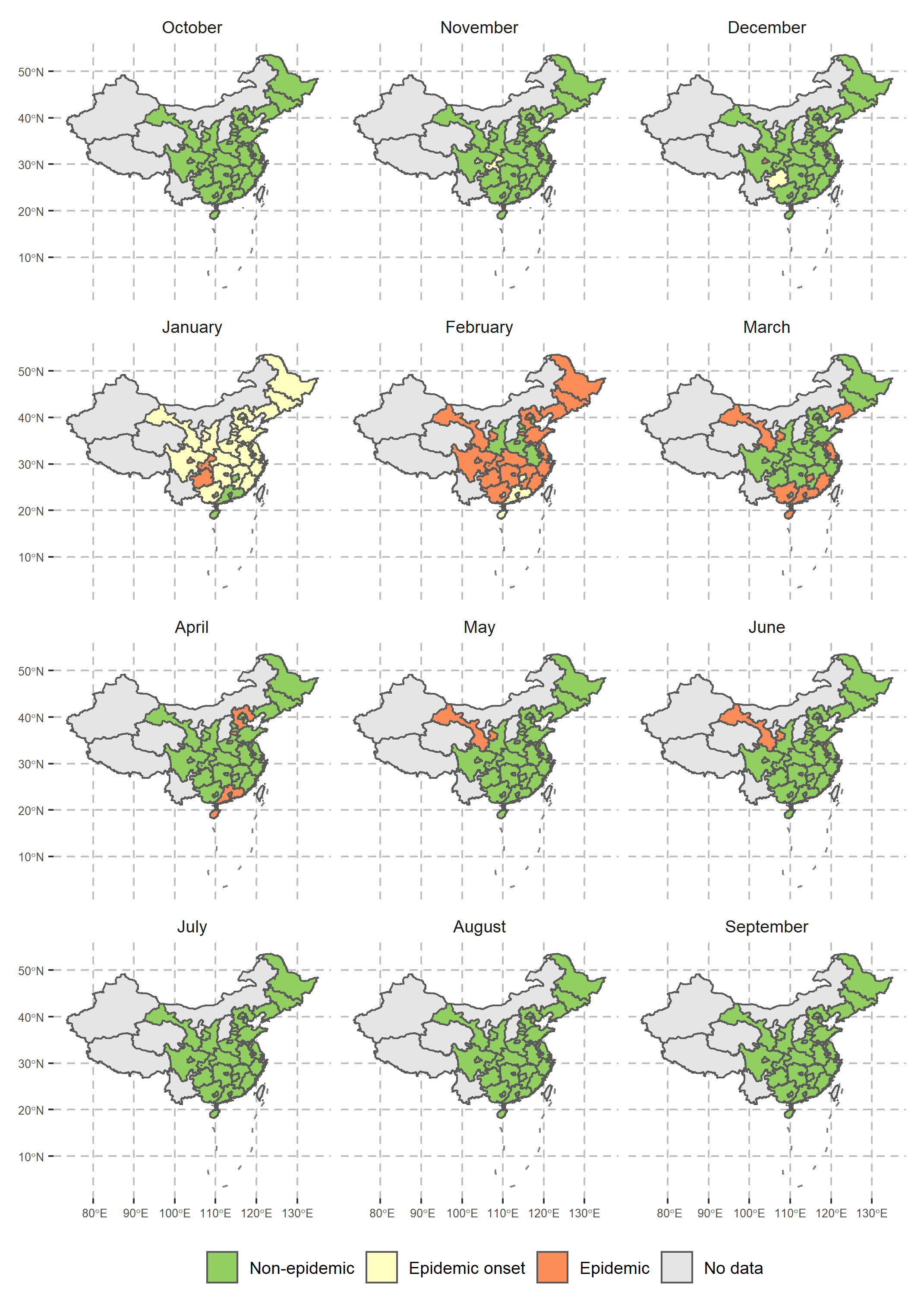


*Figure S17: Influenza A/H1N1pdm09 estimated average epidemic months across mainland China. Based on the MMR of influenza test positivity rates among ILI outpatients. Only displaying administrative regions with available data.*


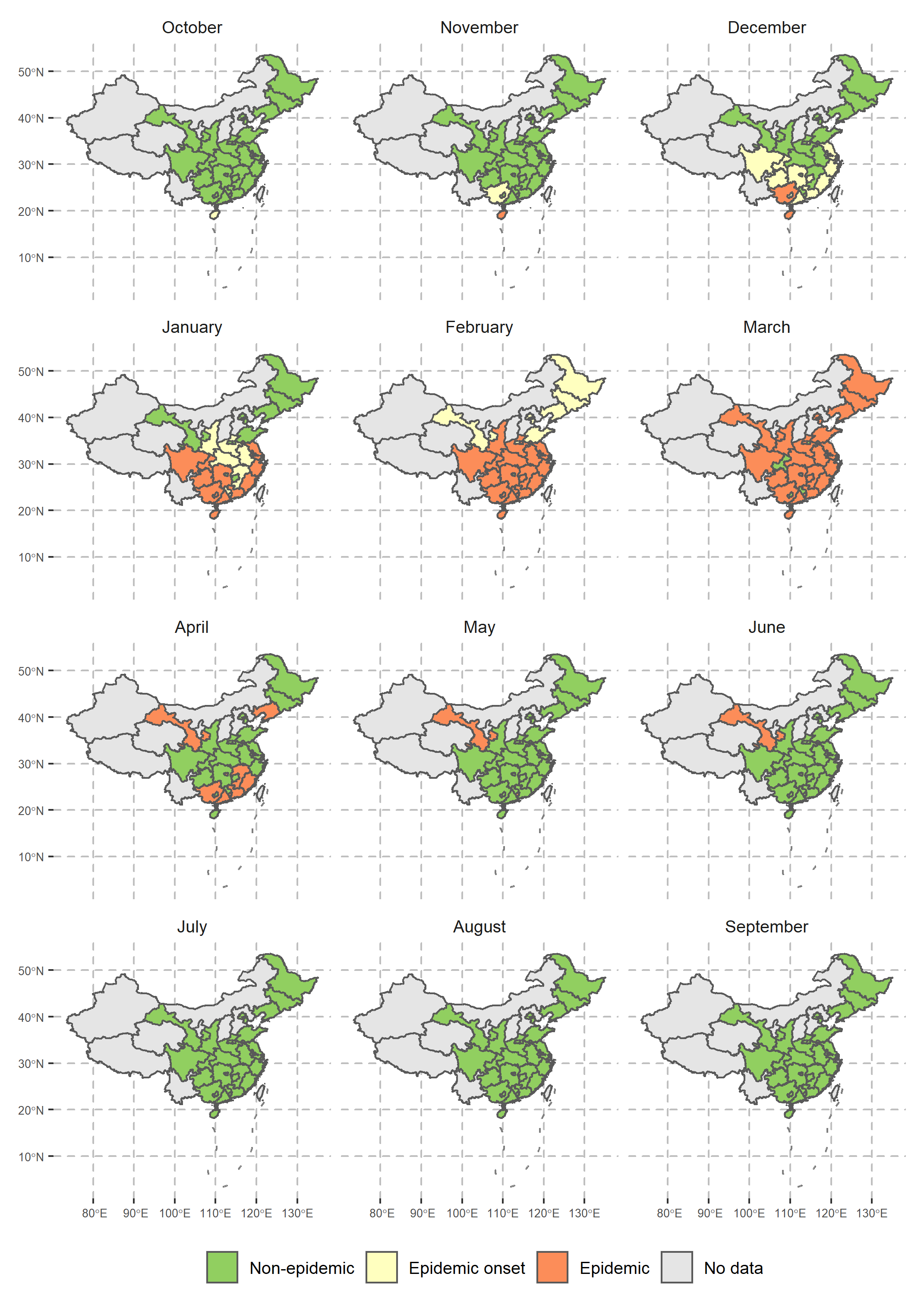


*Figure S18: Influenza B estimated average epidemic months across mainland China. Based on the MMR of influenza test positivity rates among ILI outpatients. Only displaying administrative regions with available data.*

*
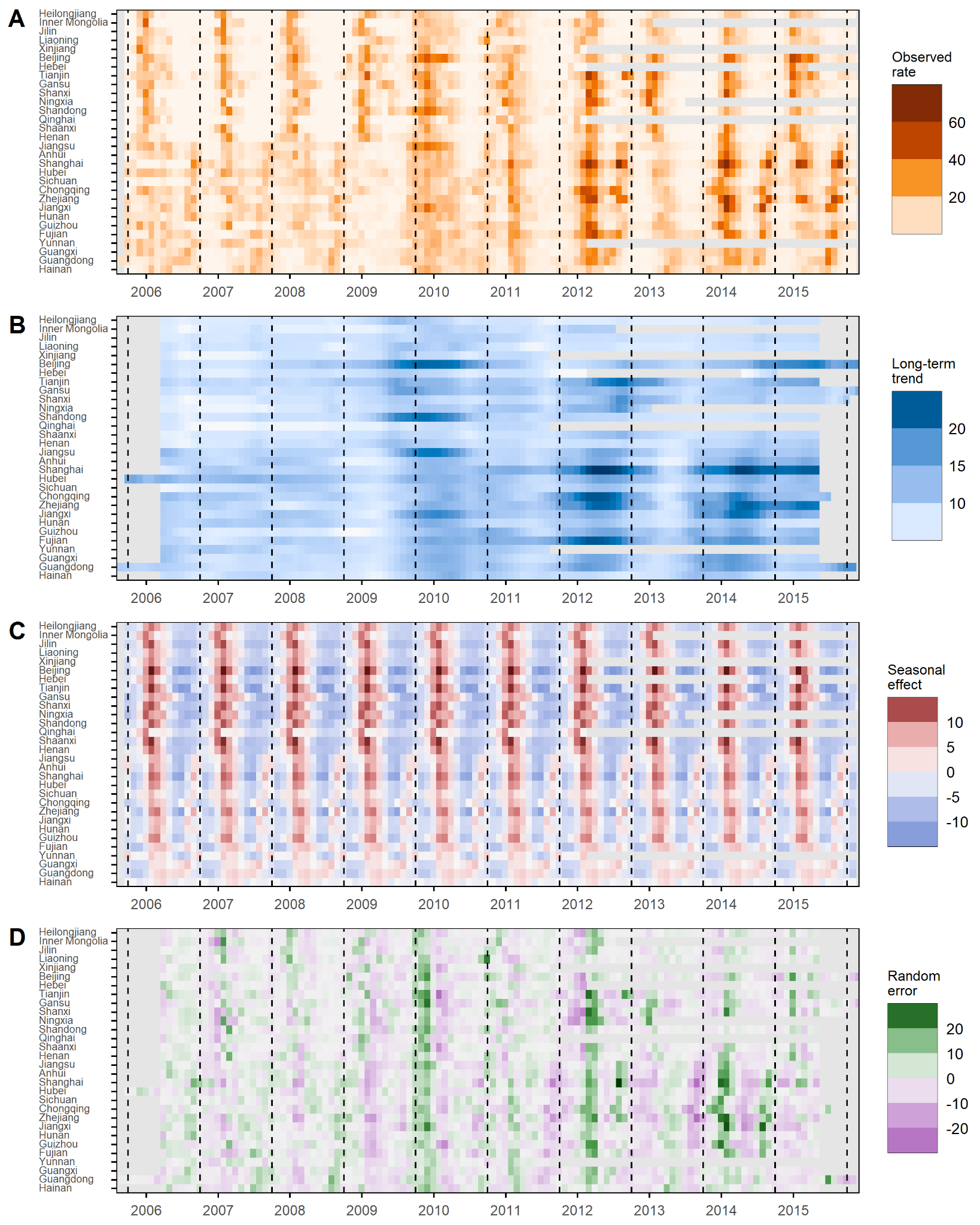
Figure S19: Seasonal decomposition of province-level all-strain influenza test positivity among ILI outpatients. A) observed rate, B) long-term trend, C) seasonal effect & D) random error. Dashed lines indicate October 1st (start of a new influenza epidemiological year). Cropped between 2006/2007 and 2015/2016 influenza seasons.*

*
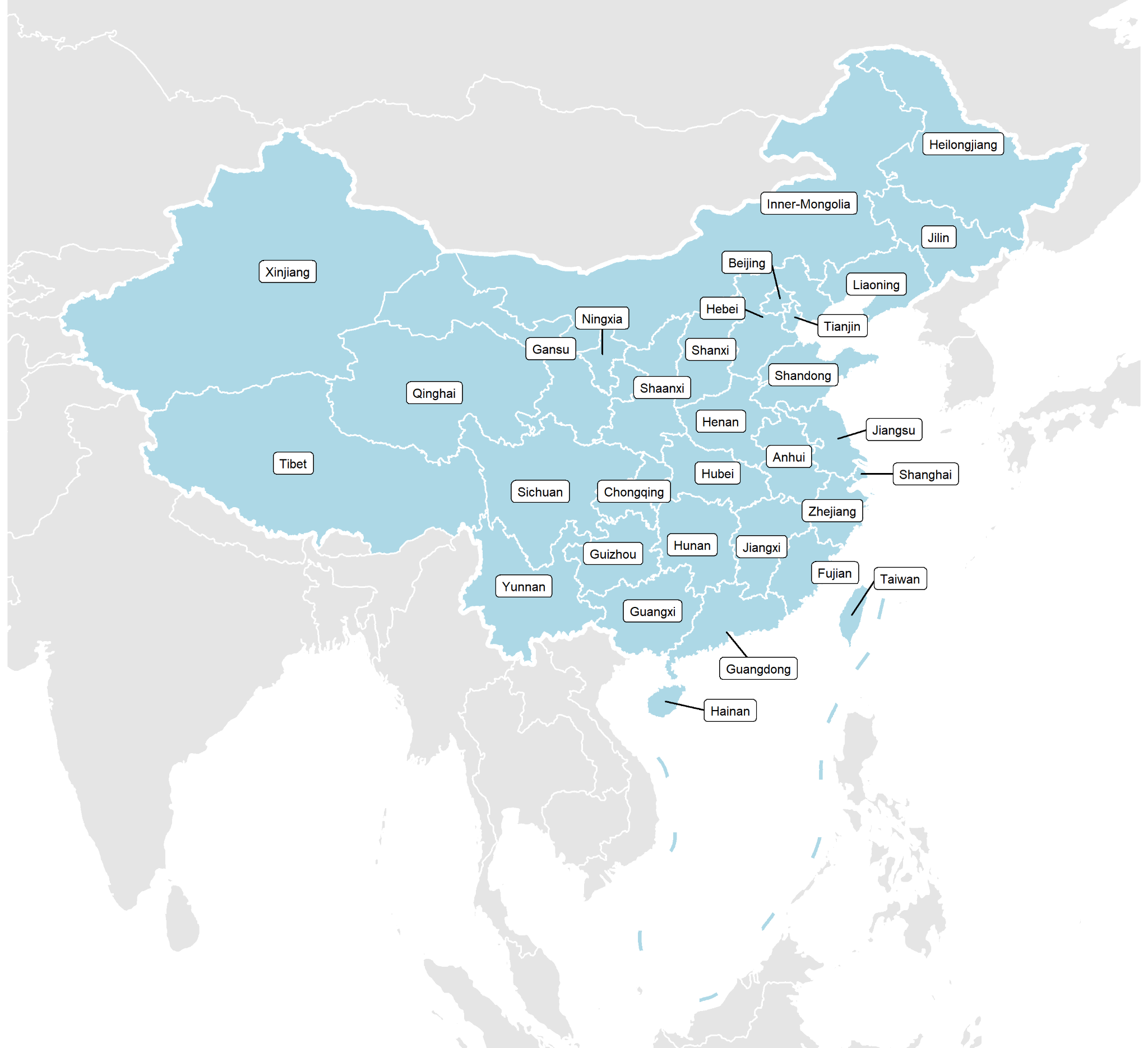
Figure S20: Province-level map of China*

| ***No.*** | ***Author*** | ***Title*** | ***Journal*** | ***Year*** |
| --- | --- | --- | --- | --- |
| 1 | 詹发先 et al., | 湖北地区2001年度流感疫情分析 | 中国病毒学 | 2002 |
| 2 | 吴海燕 et al., | 四川省2001～2004年流感监测结果分析 | 公共卫生与预防医学 | 2005 |
| 3 | 莫浩联 et al., | 2007年深圳市第四人民医院流感监测结果分析 | 热带医学杂志 | 2008 |
| 4 | 邓爱萍 et al., | 广东省2005-2007年流行性感冒流行特征分析 | 华南预防医学 | 2008 |
| 5 | Yang et al., | Review of an influenza surveillance system, Beijing, People's Republic of China | Emerg Infect Dis | 2009 |
| 6 | 刘小琦 et al., | 2006-2007年浙江省湖州市流行性感冒哨点监测结果分析 | 疾病监测 | 2009 |
| 7 | 张顺祥 et al., | 季节性流感监测中病毒分子变异分析的意义 | 中华疾病控制杂志 | 2009 |
| 8 | 陈艺韵 et al., | 2007年广州市流行性感冒病原学及血清学监测分析 | 热带医学杂志 | 2009 |
| 9 | 尤凤兴 et al., | 无锡地区流感病毒病原学监测及H3亚型血凝素基因变异研究 | 中华实验和临床病毒学杂志 | 2009 |
| 10 | 张顺祥 et al., | 深圳市2005-2007年H3N2亚型流行性感冒病毒流行病学和分子变异特征 | 中华预防医学杂志 | 2009 |
| 11 | 苏旭 et al., | 天津地区2009年流感病原学监测分析 | 中国卫生检验杂志 | 2010 |
| 12 | 邹梅 et al., | 2008—2010年大连市流感监测结果 | 职业与健康 | 2010 |
| 13 | 陆璐 et al., | 上海市卢湾区流感监测结果分析 | 中华疾病控制杂志 | 2010 |
| 14 | 周颖 et al., | 上海某哨点医院2007—2009年流感监测分析 | 上海预防医学 | 2010 |
| 15 | 孙纳 et al., | 无锡地区2008—2009年流感监测情况分析 | 职业与健康 | 2010 |
| 16 | 李翔 et al., | 宁波市H1N1型流感病毒监测分析 | 中国公共卫生 | 2010 |
| 17 | 王伟明 et al., | 2006—2009年泉州市流感监测分析 | 海峡预防医学杂志 | 2010 |
| 18 | 张锡兴 et al., | 2005-2009年长沙市流感监测结果分析 | 实用预防医学 | 2010 |
| 19 | 欧才好 et al., | 我院流感样病例监测探讨 | 中国当代医药 | 2010 |
| 20 | 林启辉 et al., | 2008～2009年流感监测结果分析 | 现代预防医学 | 2010 |
| 21 | 袁达康 et al., | 东莞市2009年流感病毒流行情况分析 | 中国卫生检验杂志 | 2010 |
| 22 | 何剑峰 et al., | 2009年广东省甲型H1N1流感流行特征分析 | 华南预防医学 | 2010 |
| 23 | 龙江 et al., | 重庆市2009-2010年流行性感冒流行特征及监测结果分析 | 热带医学杂志 | 2010 |
| 24 | 侯雪芹 et al., | 2009-2010年度广元市流感监测 | 职业卫生与病伤 | 2010 |
| 25 | 刘红雁 et al., | 玉溪市2009年流感监测分析 | 职业与健康 | 2010 |
| 26 | 刘社兰 et al., | 2009年杭州市甲型H_1N_1流感监测现状分析 | 中华临床感染病杂志 | 2010 |
| 27 | 祖荣强 et al., | 利用综合医院门诊病例数据开展呼吸道疾病症候群监测的探讨 | 中华流行病学杂志 | 2010 |
| 28 | Wang et al., | Influenza surveillance in Shenzhen, the largest migratory metropolitan city of China, 2006-2009 | Epidemiol Infect | 2011 |
| 29 | Yang et al., | Effect modification of environmental factors on influenza-associated mortality: a time-series study in two Chinese cities | BMC Infect Dis | 2011 |
| 30 | Yang et al., | Influenza associated mortality in the subtropics and tropics: results from three Asian cities | Vaccine | 2011 |
| 31 | 李凤娣 et al., | 沈阳地区2006—2009年季节性流感病原学监测分析 | 海峡预防医学杂志 | 2011 |
| 32 | 顾爽 et al., | 2008—2010年本溪市流行性感冒监测结果分析 | 职业与健康 | 2011 |
| 33 | 孙楠 et al., | 大连市2009年-2010年流感病毒核酸检测结果分析 | 中国卫生检验杂志 | 2011 |
| 34 | 杨丽华 et al., | 上海市闵行区甲型H1N1流感监测及临床相关特征分析 | 中国卫生检验杂志 | 2011 |
| 35 | 李锋平 et al., | 2009年泉州市流感监测结果分析 | 热带医学杂志 | 2011 |
| 36 | 刘娟 et al., | 烟台市2009年-2010年流感病毒检测结果分析 | 中国卫生检验杂志 | 2011 |
| 37 | 徐国锋 et al., | 2009-2010年濮阳市新甲型H1N1流感病毒检测分析 | 河南预防医学杂志 | 2011 |
| 38 | 段良松 et al., | 郴州市2006-2009年流行性感冒监测结果报告 | 实用预防医学 | 2011 |
| 39 | 梁灿坤 et al., | 珠海市2009-2010年流感防控效果评价 | 医学动物防制 | 2011 |
| 40 | 王昕 et al., | 2010年深圳市流感流行病学分析 | 热带医学杂志 | 2011 |
| 41 | 俞慕华 et al., | 2009年深圳市南山区流感病毒病原学监测结果分析 | 河南预防医学杂志 | 2011 |
| 42 | 刘艳璋 et al., | 东莞市2009年流感监测分析 | 现代预防医学 | 2011 |
| 43 | 吴丽 et al., | 来宾市514例流感病毒核酸检测结果分析 | 中国卫生检验杂志 | 2011 |
| 44 | 侯佩强 et al., | 甲型H1N1流行性感冒病毒血凝素基因变异状况及序列分析 | 中华传染病杂志 | 2011 |
| 45 | 范苏云 et al., | 深圳市福田区2007-2009年流行性感冒监测分析 | 职业与健康 | 2011 |
| 46 | Shen et al., | Epidemiologic parameters and evaluation of control measure for 2009 novel influenza a (H1N1) in Xiamen, Fujian Province, China | Virol J | 2012 |
| 47 | Guo et al., | Epidemiologic and economic burden of influenza in the outpatient setting: a prospective study in a subtropical area of China | PLoS One | 2012 |
| 48 | 齐秀荣 et al., | 2009～2011年某哨点流感监测结果分析 | 医学综述 | 2012 |
| 49 | 张晶波 et al., | 北京市西城区2009年-2011年流感流行情况分析 | 中国卫生检验杂志 | 2012 |
| 50 | 孙立新 et al., | 秦皇岛市流行性病毒性感冒疫情监测结果分析 | 职业与健康 | 2012 |
| 51 | 陆璐 et al., | 上海市卢湾区甲型H1N1流感与季节性流感流行特征比较分析 | 中华疾病控制杂志 | 2012 |
| 52 | 袁佳春 et al., | 上海市金山区2010年-2011年流感监测结果分析 | 中国卫生检验杂志 | 2012 |
| 53 | 谢晓红 et al., | 上海市奉贤区2010年流感监测情况分析 | 中国卫生检验杂志 | 2012 |
| 54 | 傅伟杰 et al., | 2009-2011年江西省流行性感冒监测分析 | 疾病监测 | 2012 |
| 55 | 王宇路 et al., | 山东省2010年-2011年流感病原学监测分析 | 中国卫生检验杂志 | 2012 |
| 56 | 杨玉芳 et al., | 2010—2011年江门市流行性感冒监测结果 | 职业与健康 | 2012 |
| 57 | 陈文青 et al., | 清远市2011年流感监测结果分析 | 中国卫生检验杂志 | 2012 |
| 58 | 曾健君 et al., | 惠州市流感病原学监测结果分析 | 热带医学杂志 | 2012 |
| 59 | 徐郁 et al., | 甲型H1N1流感流行期间珠海市流感病原学分析 | 实用预防医学 | 2012 |
| 60 | 陆剑云 et al., | 广州市2010年流行性感冒监测结果分析 | 华南预防医学 | 2012 |
| 61 | 黄振宇 et al., | 广东省东莞市流行性感冒监测系统评估 | 疾病监测 | 2012 |
| 62 | 黄海 et al., | 海口市2009~2011年流感流行特征分析 | 中国热带医学 | 2012 |
| 63 | 郎中凯 et al., | 重庆市万州区2010年-2011年流感病毒检测结果分析 | 中国卫生检验杂志 | 2012 |
| 64 | 肖达勇 et al., | 重庆市2009年流感监测结果分析 | 现代预防医学 | 2012 |
| 65 | 肖达勇 et al., | 2010年重庆市流感监测结果分析 | 预防医学情报杂志 | 2012 |
| 66 | 史映红 et al., | 绵阳市2009～2010年流感样病例哨点监测结果分析 | 现代预防医学 | 2012 |
| 67 | 孟蕾 et al., | 甘肃省2006-2011年哨点监测流感样病例动态预警分析 | 中华流行病学杂志 | 2012 |
| 68 | Yu et al., | Characterization of regional influenza seasonality patterns in China and implications for vaccination strategies: spatio-temporal modeling of surveillance data | PLoS Med | 2013 |
| 69 | Li et al., | Epidemiological analysis of respiratory viral etiology for influenza-like illness during 2010 in Zhuhai, China | Virol J | 2013 |
| 70 | Lin et al., | Influenza seasonality and predominant subtypes of influenza virus in Guangdong, China, 2004-2012 | J Thorac Dis | 2013 |
| 71 | 周大宇 et al., | 锦州市2010年-2012年甲型流感监测结果分析 | 中国卫生检验杂志 | 2013 |
| 72 | 孙佰红 et al., | 2011-2012年度辽宁省流行性感冒监测分析 | 疾病监测 | 2013 |
| 73 | 陆兵 et al., | 2005-2012年无锡市流感流行特征分析 | 中华疾病控制杂志 | 2013 |
| 74 | 杨静 et al., | 镇江市2011年-2012年流感病原学和血清学监测结果分析 | 中国卫生检验杂志 | 2013 |
| 75 | 邓婓 et al., | 江苏省2009～2011年流感监测和流行特征分析 | 现代预防医学 | 2013 |
| 76 | 雷永良 et al., | 2009~2012年丽水市流感监测网络实验室结果分析 | 中国病原生物学杂志 | 2013 |
| 77 | 杨健平 et al., | 2011-2012年赣州市流感监测结果分析 | 江西医药 | 2013 |
| 78 | 牛卫东 et al., | 郑州市2010-2012年流感病毒核酸检测结果分析 | 中国医疗前沿 | 2013 |
| 79 | 田鹏 et al., | 2010-2012年洛阳市流行性感冒监测分析 | 医学动物防制 | 2013 |
| 80 | 谢敏 et al., | 常德市2010-2012年流行性感冒病原学监测结果分析 | 实用预防医学 | 2013 |
| 81 | 叶伟雄 et al., | 2010-2011年深圳市龙岗区流感监测分析 | 职业与健康 | 2013 |
| 82 | 邹惠英 et al., | 2008—2010年深圳市龙岗区流行性感冒监测分析 | 华南预防医学 | 2013 |
| 83 | 邓星超 et al., | 2009～2012年贵港市流感监测结果分析 | 应用预防医学 | 2013 |
| 84 | 方旭东 et al., | 2007-2011年重庆市巴南区流感监测结果分析 | 预防医学情报杂志 | 2013 |
| 85 | 郭泽芊 et al., | 2009-2011年贵州省黔西南州流行性感冒监测分析 | 医学动物防制 | 2013 |
| 86 | 汤旭 et al., | 2009-2011年新疆阿克苏地区流行性感冒监测结果 | 预防医学情报杂志 | 2013 |
| 87 | Yu et al., | The substantial hospitalization burden of influenza in central China: surveillance for severe, acute respiratory infection, and influenza viruses, 2010-2012 | Influenza Other Respir Viruses | 2014 |
| 88 | Cao et al., | Forecasting influenza epidemics from multi-stream surveillance data in a subtropical city of China | PLoS One | 2014 |
| 89 | Tan et al., | Increasing similarity in the dynamics of influenza in two adjacent subtropical Chinese cities following the relaxation of border restrictions | J Gen Virol | 2014 |
| 90 | 苗芳 et al., | 2011—2012年北京市西城区哨点医院流感样病例监测结果分析 | 职业与健康 | 2014 |
| 91 | 赵冬 et al., | 2012-2013年石家庄市流行性感冒监测结果分析 | 医学动物防制 | 2014 |
| 92 | 郭卫东 et al., | 内蒙古自治区2010-2012年流感病原学及流行病学分析 | 现代预防医学 | 2014 |
| 93 | 王鑫姝 et al., | 2009-2014年本溪市儿童流感流行特征分析 | 中国临床研究 | 2014 |
| 94 | 史恒越 et al., | 2010-2014年度辽宁省阜新市流行性感冒监测结果分析 | 疾病监测 | 2014 |
| 95 | 周春芳 et al., | 上海市崇明县2012年流行性感冒监测结果分析 | 青岛医药卫生 | 2014 |
| 96 | 石平 et al., | 无锡市2010-2012年扩大流感监测结果分析 | 现代预防医学 | 2014 |
| 97 | 罗宏活 et al., | 三明市2012年流感监测及流行特点 | 海峡预防医学杂志 | 2014 |
| 98 | 周卫东 et al., | 2011～2013年吉安市流感监测分析与防控对策 | 当代医学 | 2014 |
| 99 | 杨秋峰 et al., | 河南省平顶山市2012年流感监测结果分析 | 中国热带医学 | 2014 |
| 100 | 从克 et al., | 许昌市2011年-2013年流感监测及病毒流行特征分析 | 中国卫生检验杂志 | 2014 |
| 101 | 马莹莹 et al., | 河南省许昌市2009-2012年流感监测结果分析 | 现代预防医学 | 2014 |
| 102 | 李月 et al., | 随州市2010-2013年哨点医院流感监测 | 公共卫生与预防医学 | 2014 |
| 103 | 陈文青 et al., | 清远市流行性感冒病原学监测分析 | 热带医学杂志 | 2014 |
| 104 | 苏雪銮 et al., | 2013年广东省潮州市流行性感冒流行特征分析 | 分子诊断与治疗杂志 | 2014 |
| 105 | 曹文萍 et al., | 2011~2013年成都市某哨点医院流感监测结果分析 | 热带病与寄生虫学 | 2014 |
| 106 | 张翠萍 et al., | 武威市2009~2012年流感样病例监测结果分析 | 现代生物医学进展 | 2014 |
| 107 | 张玉霞 et al., | 2011—2013年定西市流感监测结果分析 | 卫生职业教育 | 2014 |
| 108 | 李红育 et al., | 甘肃省2009年-2012年流感监测及流行特征分析 | 中国卫生检验杂志 | 2014 |
| 109 | 于德山 et al., | 甘肃省2009—2012年流感病毒流行特征分析 | 中国病毒病杂志 | 2014 |
| 110 | 范苏云 et al., | 2010-2012年深圳市福田区流感监测分析 | 预防医学情报杂志 | 2014 |
| 111 | Wang et al., | Using an adjusted Serfling regression model to improve the early warning at the arrival of peak timing of influenza in Beijing | PLoS One | 2015 |
| 112 | Fu et al., | The clinical and etiological characteristics of influenza-like illness (ILI) in outpatients in Shanghai, China, 2011 to 2013 | PLoS One | 2015 |
| 113 | Li et al., | Interim estimates of divergence date and vaccine strain match of human influenza A(H3N2) virus from systematic influenza surveillance (2010-2015) in Hangzhou, southeast of China | Int J Infect Dis | 2015 |
| 114 | 孙伟明 et al., | 2009-2014年邢台市新型甲型H1N1流感病原学监测分析 | 现代预防医学 | 2015 |
| 115 | 王冰 et al., | 沈阳市2013年-2014年流感监测结果分析 | 中国卫生检验杂志 | 2015 |
| 116 | 杨吉星 et al., | 2009-2013年度上海市虹口区流行性感冒监测分析 | 热带医学杂志 | 2015 |
| 117 | 李芳 et al., | 2012-2013年上海市普陀区流感病原学分析 | 现代预防医学 | 2015 |
| 118 | 雍玮 et al., | 2014年南京市流感病毒监测结果分析 | 江苏预防医学 | 2015 |
| 119 | 魏叶 et al., | 南通市2011-2014年流感样病例和病原学监测分析 | 医学动物防制 | 2015 |
| 120 | 葛海燕 et al., | 2013-2014年淮安市某哨点医院流行性感冒监测结果分析 | 东南国防医药 | 2015 |
| 121 | 朱伟光 et al., | 2011年-2013年宿迁市流感病毒病原学监测分析 | 中国卫生检验杂志 | 2015 |
| 122 | 邵铁娟 et al., | 2009～2013年杭州地区甲型流感病毒基质蛋白基因的遗传进化分析 | 病毒学报 | 2015 |
| 123 | 林丽莲 et al., | 漳州市2011—2013年流感流行特征分析 | 海峡预防医学杂志 | 2015 |
| 124 | 赖建萍 et al., | 2009-2012年鹰潭市流行性感冒监测分析 | 现代预防医学 | 2015 |
| 125 | 杨跃进 et al., | 2012-2013年开封市流行性感冒监测结果分析 | 华南预防医学 | 2015 |
| 126 | 李岩 et al., | 2009-2015年河北省流感病原学监测结果分析 | 中国病原生物学杂志 | 2015 |
| 127 | 刘琳琳 et al., | 湖北省2006-2012年度流行性感冒监测结果分析 | 中华疾病控制杂志 | 2015 |
| 128 | 李晓明 et al., | 株洲市2008-2013年流行性感冒监测结果分析 | 实用预防医学 | 2015 |
| 129 | 李铁钢 et al., | 新型甲型H1N1流感监测体系的建立与应用分析 | 热带医学杂志 | 2015 |
| 130 | 黄涛 et al., | 2013年崇左市流行性感冒实验室检测结果分析 | 职业与健康 | 2015 |
| 131 | 谭珍连 et al., | 2010-2013年梧州市流感流行特征分析 | 现代预防医学 | 2015 |
| 132 | 史映红 et al., | 2010-2013年绵阳市流行性感冒监测分析 | 华南预防医学 | 2015 |
| 133 | 温秋芳 et al., | 2009年-2013年宁夏流感病原学监测分析 | 中国卫生检验杂志 | 2015 |
| 134 | 华伟玉 et al., | 北京市海淀区哨点医院流感样病例监测资料研究 | 国际病毒学杂志 | 2015 |
| 135 | 王秉谦 et al., | 天津市红桥区2012年～2014年哨点医院流感样病例流行情况分析 | 医学信息 | 2015 |
| 136 | 李岩 et al., | 河北省2009-2014年甲型H1N1流感流行特征分析 | 国际病毒学杂志 | 2015 |
| 137 | 戴文军 et al., | 2013年泰州市门诊急性上呼吸道感染监测结果分析 | 中国校医 | 2015 |
| 138 | Wu et al., | Coherence of Influenza Surveillance Data across Different Sources and Age Groups, Beijing, China, 2008-2015 | PLoS One | 2016 |
| 139 | Liu et al., | Dynamic patterns of circulating influenza virus from 2005 to 2012 in Shandong Province, China | Arch Virol | 2016 |
| 140 | Guo et al., | Impact of Influenza on Outpatient Visits, Hospitalizations, and Deaths by Using a Time Series Poisson Generalized Additive Model | PLoS One | 2016 |
| 141 | Qi et al., | Epidemiological and Virological Characteristics of Influenza in Chongqing, China, 2011-2015 | PLoS One | 2016 |
| 142 | 庞晶晶 et al., | 2014年北京市西城区流感监测结果分析 | 职业与健康 | 2016 |
| 143 | 刘国良 et al., | 2013年-2015年廊坊市流感病原学监测分析 | 中国卫生检验杂志 | 2016 |
| 144 | 韩光跃 et al., | 河北省2014年-2015年流感监测结果分析 | 中国卫生检验杂志 | 2016 |
| 145 | 张瑞卿 et al., | 2012年-2016年晋中市流感监测结果分析 | 临床检验杂志(电子版) | 2016 |
| 146 | 徐萌杰 et al., | 2009年-2015年鄂尔多斯市流感监测结果分析 | 中国卫生检验杂志 | 2016 |
| 147 | 孙佰红 et al., | 辽宁省2010—2014年流感流行特征及变化趋势 | 中国公共卫生 | 2016 |
| 148 | 陈诹 et al., | 上海市浦东新区2012—2014年流行性感冒病原学监测 | 中国热带医学 | 2016 |
| 149 | 钱程 et al., | 2012—2014年江阴市流感监测结果分析 | 职业与健康 | 2016 |
| 150 | 张潇丹 et al., | 2015镇江市流感病原学监测结果分析 | 中国卫生检验杂志 | 2016 |
| 151 | 罗锋 et al., | 2015年泰州市流感样病例病原谱及流行病学特征 | 江苏预防医学 | 2016 |
| 152 | 吴照春 et al., | 2015—2016年度安庆市流行性感冒监测分析 | 职业与健康 | 2016 |
| 153 | 吴照春 et al., | 2010-2014年安庆市流行性感冒监测结果分析 | 疾病监测 | 2016 |
| 154 | 郑雯菱 et al., | 南平市2011年-2015年流行性感冒监测结果分析 | 中国卫生检验杂志 | 2016 |
| 155 | 廖亦红 et al., | 龙岩市2012-2014年流感监测分析 | 中国公共卫生管理 | 2016 |
| 156 | 贺凤兰 et al., | 2011年-2015年南昌市流感监测哨点医院病原学检测结果分析 | 中国卫生检验杂志 | 2016 |
| 157 | 刘娟 et al., | 2010年-2014年烟台流感病原学监测分析 | 中国卫生检验杂志 | 2016 |
| 158 | 项爱红 et al., | 汤阴县2015年流感病原学监测结果分析 | 中国卫生检验杂志 | 2016 |
| 159 | 于燕 et al., | 2010-2015年河南省哨点医院流感样病例监测分析 | 现代预防医学 | 2016 |
| 160 | 代丽丽 et al., | 2010-2014年湖北省荆门市流感哨点监测结果分析 | 职业卫生与病伤 | 2016 |
| 161 | 刘琳琳 et al., | 湖北省2010～2014年度流行性感冒流行态势分析 | 华中科技大学学报(医学版) | 2016 |
| 162 | 王中秋 et al., | 湘潭市2014年-2015年流感病原学监测结果分析 | 中国卫生检验杂志 | 2016 |
| 163 | 彭力荇 et al., | 2010-2014年云浮市流行性感冒病原学监测分析 | 实用预防医学 | 2016 |
| 164 | 王顺东 et al., | 2010-2014年达州市流感监测结果分析 | 现代预防医学 | 2016 |
| 165 | 周丽君 et al., | 2011-2014年四川省流行性感冒监测结果分析 | 职业卫生与病伤 | 2016 |
| 166 | 刘方遥 et al., | 2011-2015年北京市海淀区流感监测结果分析 | 国际病毒学杂志 | 2016 |
| 167 | 乔鹏 et al., | 上海市杨浦区2010-2015年哨点医院儿童流感监测结果 | 上海预防医学 | 2016 |
| 168 | 陈维 et al., | 舟山市某院严重急性呼吸道感染病例流行特点分析 | 上海预防医学 | 2016 |
| 169 | 王昕 et al., | 广东省深圳市流感流行特征分析 | 国际病毒学杂志 | 2016 |
| 170 | 李保娣 et al., | 2013-2015年度甘肃省流感病毒病原学特征分析 | 中华实验和临床病毒学杂志 | 2016 |
| 171 | 杨筱婷 et al., | 甘肃省流感流行预警方法探研 | 中华流行病学杂志 | 2016 |
| 172 | Wu et al., | Estimated incidence and number of outpatient visits for seasonal influenza in 2015-2016 in Beijing, China | Epidemiol Infect | 2017 |
| 173 | Yu et al., | Excess pneumonia and influenza mortality attributable to seasonal influenza in subtropical Shanghai, China | BMC Infect Dis | 2017 |
| 174 | Wang et al., | Epidemiological Features and Forecast Model Analysis for the Morbidity of Influenza in Ningbo, China, 2006-2014 | Int J Environ Res Public Health | 2017 |
| 175 | Cheng et al., | Comparison of Influenza Epidemiological and Virological Characteristics between Outpatients and Inpatients in Zhejiang Province, China, March 2011-June 2015 | Int J Environ Res Public Health | 2017 |
| 176 | Liu et al., | Seasonal pattern of influenza activity in a subtropical city, China, 2010-2015 | Sci Rep | 2017 |
| 177 | Liu et al., | Excess mortality associated with influenza after the 2009 H1N1 pandemic in a subtropical city in China, 2010-2015 | Int J Infect Dis | 2017 |
| 178 | 耿利彬 et al., | 2011—2016年北京市密云区流感监测分析 | 公共卫生与预防医学 | 2017 |
| 179 | 温雯 et al., | 2015-2016年北京市朝阳区流感流行特征及病原学分析 | 中华疾病控制杂志 | 2017 |
| 180 | 李岩 et al., | 石家庄地区2014-2016年甲型H1N1流感病原学及血清学监测分析 | 中国病毒病杂志 | 2017 |
| 181 | 韩光跃 et al., | 河北省2011-2017年甲型H1N1流感流行及其基因特征分析 | 中国病毒病杂志 | 2017 |
| 182 | 王静 et al., | 2009—2017年晋城市流行性感冒病原学监测结果分析 | 长治医学院学报 | 2017 |
| 183 | 高美琴 et al., | 鄂尔多斯市2016年流感监测结果分析 | 中国卫生检验杂志 | 2017 |
| 184 | 王慧馨 et al., | 2013-2015年内蒙古自治区流行性感冒流行病学特征与病原学分析 | 中国病毒病杂志 | 2017 |
| 185 | 李航 et al., | 2015至2016年度丹东市流感病原学监测结果分析 | 沈阳医学院学报 | 2017 |
| 186 | 李翠珍 et al., | 2013—2016年上海市闵行区流感监测结果分析 | 复旦学报(医学版) | 2017 |
| 187 | 王炜翔 et al., | 南京市2006-2015年流感流行病学特征分析 | 中华疾病控制杂志 | 2017 |
| 188 | 罗宏活 et al., | 三明市2013—2015年流行性感冒监测分析 | 海峡预防医学杂志 | 2017 |
| 189 | 王伟 et al., | 2012-2015年洛阳市流感监测结果分析 | 河南预防医学杂志 | 2017 |
| 190 | 李巧玲 et al., | 2015-2016年湘潭市流行性感冒监测结果分析 | 河南预防医学杂志 | 2017 |
| 191 | 陈艺韵 et al., | 广州市2012—2016年A型季节性流感的流行特征分析 | 中国热带医学 | 2017 |
| 192 | 农皓 et al., | 南宁市2012—2015年流行性感冒流行特征分析 | 微生物学免疫学进展 | 2017 |
| 193 | 朱向晖 et al., | 核酸检测在流感防控中的应用研究 | 疾病监测与控制 | 2017 |
| 194 | 张俊君 et al., | 2015-2017年陕西省咸阳市流行性感冒流行特征分析 | 医学动物防制 | 2017 |
| 195 | 许晶 et al., | 2010-2016年陕西省度流行性感冒监测分析 | 现代预防医学 | 2017 |
| 196 | 房小梅 et al., | 武威市2011-2016年流感病原学监测分析 | 中国公共卫生管理 | 2017 |
| 197 | 孔席丽 et al., | 2009—2017年新疆巴州流感监测分析 | 疾病预防控制通报 | 2017 |
| 198 | Wang et al., | Using a community based survey of healthcare seeking behavior to estimate the actual magnitude of influenza among adults in Beijing during 2013-2014 season | BMC Infect Dis | 2017 |
| 199 | 王小莉 et al., | 基于气象及流感病毒活动度的流感流行预测研究 | 国际病毒学杂志 | 2017 |
| 200 | 李岩 et al., | 2011-2017年河北省甲型H1N1流感流行特征分析 | 国际病毒学杂志 | 2017 |
| 201 | 刘玲 et al., | 山西省2011-2016年流感病原学监测结果分析 | 国际病毒学杂志 | 2017 |
| 202 | 李翠珍 et al., | 2013-2016年上海市闵行区流感监测结果分析 | 复旦学报（医学版) | 2017 |
| 203 | 杨丽华 et al., | 上海市松江地区急性上呼吸道感染患儿呼吸道病毒感染研究 | 国际病毒学杂志 | 2017 |
| 204 | 崔一敏 et al., | 2012——2016年黔江区流感流行特征分析 | 中国保健营养 | 2017 |
| 205 | 杨筱婷 et al., | 甘肃省流感季节性流行特征及病原变迁研究 | 中华流行病学杂志 | 2017 |
| 206 | Cheng et al., | Using an innovative method to develop the threshold of seasonal influenza epidemic in China | PLoS One | 2018 |
| 207 | Zhang et al., | Influenza-associated mortality in Yancheng, China, 2011-15 | Influenza Other Respir Viruses | 2018 |
| 208 | 李若曦 et al., | ARIMA模型在北京市丰台区流行性感冒预测中的应用 | 职业与健康 | 2018 |
| 209 | 卢达 et al., | 2010-2015年天津市河东区流行性感冒监测分析 | 河南预防医学杂志 | 2018 |
| 210 | 王英 et al., | 天津市滨海新区2014年-2017年流感病原学特征分析 | 中国卫生检验杂志 | 2018 |
| 211 | 周景 et al., | 南京市儿童医院2013—2017年流感流行病学特征分析 | 东南大学学报(医学版) | 2018 |
| 212 | 颜雅娟 et al., | 2012-2017年江阴市流感监测结果分析 | 现代预防医学 | 2018 |
| 213 | 邹娇娇 et al., | 2011—2016年武汉市流感样病例监测结果及预测分析 | 应用预防医学 | 2018 |
| 214 | 何秋瑜 et al., | 广东省梅州市2011年-2016年流感病原学监测结果分析 | 中国卫生检验杂志 | 2018 |
| 215 | 叶俊凯 et al., | 广州地区某哨点医院2013-2016年度流行性感冒流行态势 | 热带医学杂志 | 2018 |
| 216 | 张科 et al., | 重庆市渝中区2013—2016年流感哨点监测结果分析 | 现代医药卫生 | 2018 |
| 217 | 孙彦峰 et al., | 2010-2017年宝鸡市流感样病例病原学检测结果分析 | 现代预防医学 | 2018 |
| 218 | 马文瑛 et al., | 甘肃省敦煌市2009年8月至2018年6月流行性感冒病原学监测结果分析 | 中国病毒病杂志 | 2018 |
| 219 | 蒲玉娇 et al., | 新疆乌鲁木齐市2011年-2015年流感监测分析 | 中国卫生检验杂志 | 2018 |
| 220 | 张璇 et al., | 2013—2016年新疆流感病原学监测结果 | 职业与健康 | 2018 |
| 221 | 苏健婷 et al., | 北京市2007—2013年季节性流感和2009年甲型H1N1流感大流行的死亡负担研究 | 国际病毒学杂志 | 2018 |
| 222 | Dai et al., | The effect of ambient temperature on the activity of influenza and influenza like illness in Jiangsu Province, China | Sci. Total Environ | 2018 |
| 223 | 康敏 et al., | 广东省2017-2018年冬季流感流行特征研究 | 中华流行病学杂志 | 2018 |
| 224 | Su et al., | City-Wide Influenza Forecasting based on Multi-Source Data | IEEE | 2018 |
| 225 | Li et al., | Influenza-associated excess respiratory mortality in China, 2010-15: a population-based study | Lancet Public Health | 2019 |
| 226 | Ye et al., | Understanding the complex seasonality of seasonal influenza A and B virus transmission: Evidence from six years of surveillance data in Shanghai, China | Int J Infect Dis | 2019 |
| 227 | Su et al., | Forecasting influenza activity using self-adaptive AI model and multi-source data in Chongqing, China | EBioMedicine | 2019 |
| 228 | Pan et al., | Association of meteorological factors with seasonal activity of influenza A subtypes and B lineages in subtropical western China | Epidemiol Infect | 2019 |
| 229 | Zhou et al., | Temporal patterns of influenza A subtypes and B lineages across age in a subtropical city, during pre-pandemic, pandemic, and post-pandemic seasons | BMC Infect Dis | 2019 |
| 230 | 郑兰紫 et al., | 2012—2018年北京市密云区流感病原学监测分析 | 公共卫生与预防医学 | 2019 |
| 231 | 石华 et al., | 2017年度大同市流感病毒病原学特征分析 | 预防医学论坛 | 2019 |
| 232 | 王唐 et al., | 上海市金山区2015-2017年流感监测结果分析 | 实用预防医学 | 2019 |
| 233 | 王静 et al., | 上海市浦东新区2015-2017年流感监测结果分析 | 实用预防医学 | 2019 |
| 234 | 刘晓骏 et al., | 江苏省江阴市2014-2016年乙型流感病毒流行特征分析 | 医学动物防制 | 2019 |
| 235 | 杨焕森 et al., | 2016—2017年连云港市流感监测分析 | 中国校医 | 2019 |
| 236 | 许纯 et al., | 扬州市2017年流感哨点医院监测结果 | 江苏预防医学 | 2019 |
| 237 | 唐光媛 et al., | 2014-2017年镇江市流感流行病学特征 | 江苏预防医学 | 2019 |
| 238 | 陈步青 et al., | 2012年-2017年浙江省义乌市流感病原学监测分析 | 中国卫生检验杂志 | 2019 |
| 239 | 邓兰 et al., | 2012-2017年江西省萍乡市流感病原学监测结果分析 | 现代预防医学 | 2019 |
| 240 | 吴杰 et al., | 滨州市流行性感冒监测结果分析 | 预防医学 | 2019 |
| 241 | 栾旭波 et al., | 2016-2018年新乡市监测哨点流感样病例结果分析 | 河南预防医学杂志 | 2019 |
| 242 | 贾桂华 et al., | 2017-2018年许昌市流感样病例病原学监测结果分析 | 河南预防医学杂志 | 2019 |
| 243 | 候少华 et al., | 湖北省咸宁市2013-2017年流感监测结果分析 | 实用预防医学 | 2019 |
| 244 | 丁小满 et al., | 深圳市南山区流感流行特征分析 | 中国热带医学 | 2019 |
| 245 | 吴林杰 et al., | 杭州地区2017年呼吸道感染2337例病原体检测结果分析 | 中国乡村医药 | 2019 |
| 246 | 钱亚琼 et al., | 甲型和乙型流感病毒抗原快速检测结果分析 | 浙江临床医学 | 2019 |
| 247 | 周雯 et al., | 2016-2018年北海市流感样病例监测情况分析 | 健康之友 | 2019 |

*Table S1: Final studies included in quantitative synthesis after title, abstract and full text screening.*
